# Mechanism of Action of the Sesquiterpene Compound Helenalin in Rhabdomyosarcoma Cells

**DOI:** 10.3390/ph14121258

**Published:** 2021-12-02

**Authors:** Hakmin Mun, Helen Elizabeth Townley

**Affiliations:** 1 Nuffield Department of Women’s and Reproductive Health, University of Oxford, John Radcliffe Hospital, Oxford OX3 9DU, UK; hak.mun@some.ox.ac.uk; 2 Department of Engineering Science, University of Oxford, Parks Road, Oxford OX1 3PJ, UK

**Keywords:** sesquiterpene lactone, helenalin, rhabdomyosarcoma, mechanism of action, anti-tumour activity, antagonism

## Abstract

Rhabdomyosarcoma (RMS) is the most frequent soft tissue sarcoma in paediatric patients. Relapsed or refractory RMS shows very low 5-year survival rates, which urgently necessitates new chemotherapy agents. Herein, the sesquiterpene lactone, helenalin, was investigated as a new potential therapeutic agent against the embryonal RMS (eRMS) and alveolar RMS (aRMS) cells. We have evaluated in vitro antiproliferative efficacy of helenalin on RMS cells by the MTT and wound healing assay, and estimated several cell death pathways by flow cytometry, confocal microscopy and immunoblotting. It was shown that helenalin was able to increase reactive oxygen species levels, decrease mitochondrial membrane potential, trigger endoplasmic reticulum stress and deactivate the NF-**κ**B pathway. Confirmation was obtained through the use of antagonistic compounds which alleviated the effects of helenalin in the corresponding pathways. Our findings demonstrate that oxidative stress is the pivotal mechanism of action of helenalin in promoting RMS cell death in vitro.

## 1. Introduction

RMS is the most frequent soft tissue sarcoma of childhood and adolescence, accounting for up to 40% of paediatric sarcomas [1]. RMS is mainly classified into two subtypes, i.e., embryonal RMS (eRMS) and alveolar RMS (aRMS), which have significant differences with respect to their genetics, prognosis and survival rate [2]. eRMS normally exhibits allelic loss at chromosome 11p15.5, whose loss-of-function can result in activation of oncogenes (e.g., IGF2, HRAS) and deactivation of tumour suppressing genes (e.g., H19, CDKN1C) [3]. Chromosomal translocations such as t(2;13)(q35;q14) and t(1;13)(q36;q14) are the main features of the aRMS genome [4]. The reciprocal translocations in chromosomes lead to a fusion between the PAX3 and FOXO1 gene, or between the PAX7 with FOXO1 gene, and those fusion genes predominantly express many essential proteins, i.e., PAX3-FOXO1 or PAX7-FOXO1, that are able to activate crucial oncogenes (e.g., MYC, MYCN) through interactions with super enhancers [5]. The 5-year survival rates of eRMS and aRMS are approximately 75% and less than 50% respectively; relapsed or refractory RMS displays poorer prognosis with survival rates between 10% and 30% [6].

Helenalin, a sesquiterpene lactone, is a secondary metabolite predominantly originating from flowering plants such as *Arnica montana* and *Arnica chamissonis* ssp. *Foliosa* [7]. Helenalin possesses two alkylating centres which are based on α, β- unsaturated carbonyl structures of α- methylene-ɣ-lactone and a cyclopentenone moiety. These alkylating centres are capable of interacting with bionucleophiles, e.g., sulfhydryl-bearing enzymes, through a Michael reaction [8]. The alkylation competence of helenalin is directly or indirectly associated with inhibition of DNA polymerase and protein synthesis [9], telomerase [10], glutathione and cysteine levels [11] in many cells. Nuclear factor kappa-light-chain-enhancer of activated B cells (NF-**κ**B) is a well-known protein suppressively controlled by helenalin, even though there are a number of potential molecular targets for helenalin [12]. Helenalin has previously been reported to modify the NF-**κ**B/Inhibitor of kappa B (I**κ**B) and prevent the release of NF-**κ**B dimers, which can translocate into the nucleus and bind to numerous **κ**B sites (the consensus sequence GGGRNNYYCC; where R: purine, Y: pyrimidine, N: any base) and activate genes related to cell proliferation, cell survival, metastasis and angiogenesis [7,13,14].

The antineoplastic potency of helenalin has been determined in vitro and in vivo against other cancers such as leukaemia [15], breast and renal carcinoma [16,17] and glioma cells [18], but the impact of helenalin on paediatric cancer cell lines such as RMS has not been documented. Due to their origin, paediatric cancers are known to differ from adult cancers in both their speed of growth and susceptibility to particular chemotherapy agents, amongst other factors. Helenalin has been shown to induce apoptosis in doxorubicin-resistant tumour cells by decreasing mitochondrial membrane potential (MMP, Δ𝚿m) and downregulating PI3K/AKT/m-TOR signalling pathway [19]. The inhibition of NF-**κ**B p65 expression appears to be a fundamental mechanism of helenalin in inducing autophagy in tumour cells by increasing the levels of autophagic enzymatic markers, i.e., Atg12 and LC3-B, and triggering the cleavage of Caspase 3 and 9. The reliance on NF-**κ**B for helenalin-induced cell death has been confirmed through the exogenous overexpression of NF-**κ**B p65 in tumour cells, which resulted in the reduction of caspase cleavage and tumour cell death [16]. Some investigations have claimed that helenalin was able to alleviate oxidative stress and reduce ROS levels by activating the Nrf2 signalling pathway, contributing to the attenuation of cellular apoptosis [20].

Although a number of studies have revealed several roles of helenalin, e.g., mitochondrial dissipation, ROS production and NF-**κ**B deactivation, in eliciting cytotoxicity against various tumour cells, the main anti-tumour mechanism of action has not been fully elucidated. Therefore, this research aimed to find out the dominant cellular pathway in helenalin-induced RMS cell death by employing antagonistic compounds to several mechanisms and assessing the degree of cell survival. In this study, we investigated the cytotoxic effect of helenalin on two RMS cell lines, RD (eRMS) and RH30 (aRMS) and examined the oxidative stress, mitochondrial depolarisation, endoplasmic reticulum (ER) stress and NF-**κ**B inhibition pathways; as a result, we identified that the oxidative stress from ROS generation induced by helenalin might be the primary action of helenalin towards RMS cell death.

## 2. Results

### 2.1. Cytotoxicity of Helenalin against RMS Cells

**Figure 1:**
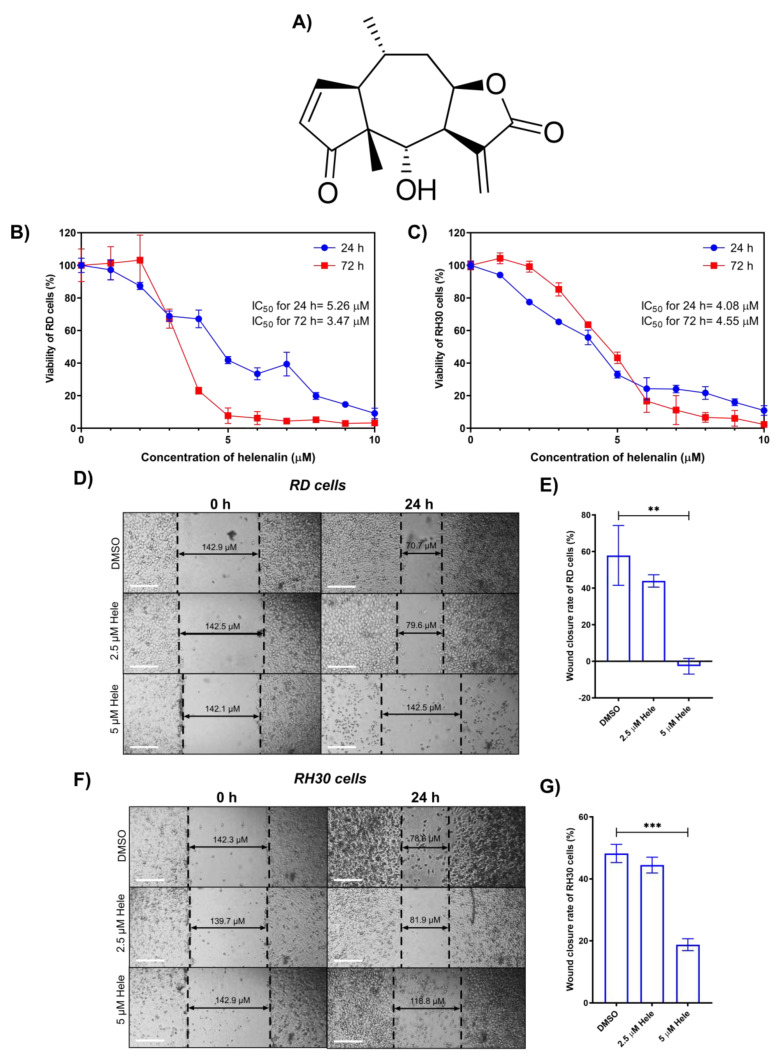
Effect of helenalin on RMS cell growth. (**A**) Chemical structure of helenalin. (**B**,**C**) Viable cell number assessed using an MTT assay after cells were treated with various concentrations (0–10 µM) of helenalin (Hele) for 24 h and 72 h in RD and RH30 cells. (**D**,**F**) In vitro wound healing assay of RD and RH30 cells after treating cells with drugs for 24 h, (Scale bars = 50 µm). (**E**,**G**) Quantification of the wound closure rate of RD and RH30 cells upon the drug treatment evaluated from the wound healing assays. Significances were tested using one-way analysis of variance (ANOVA) with Dunnett post hoc tests (** *p* ≤ 0.01, *** *p* ≤ 0.001).

The antiproliferative effect of helenalin (Figure 1A) was evaluated in RD and RH30 cells by the MTT (3-(4,5-dimethylthiazol-2-yl)-2,5-diphenyltetrazolium bromide) assay after treatment with helenalin for 24 h and 72 h. Helenalin showed a decrease in cell numbers in a dose-dependent manner. RD cells showed an IC_50_ of 5.26 µM for 24 h treatment and 3.47 µM for 72 h treatment, which indicated that helenalin is able to induce the RD cell death in a time- and dose-dependent manner (Figure 1B). RH30 cells were shown to be more sensitive (IC_50_ = 4.08 µM) to helenalin than RD cells during the 24 h period, whereas RH30 cells were less susceptible (IC_50_ = 4.55 µM) to helenalin than RD cells during the 72 h period (Figure 1C). This outcome demonstrated that RH30 cells are likely to be more resistant to chemotherapeutic agents than RD cells during long-time exposure, since the former is a metastatic tumour line, and the latter is not. Moreover, RH30 cells had a higher IC_50_ at 72 h than that at 24 h, which indicated that helenalin had a reduced cytotoxicity in RH30 cells after prolonged treatment. This may be due to the development of chemoresistance in the cells, or that helenalin is unstable and loses its potency over time. Consequently, the stability of helenalin was evaluated by incubation in phosphate-buffered saline (PBS) at 37 °C in a 5% CO_2_ atmosphere for 3 days (72 h). Indeed, it was found that helenalin degraded over time, such that only 68.2 ± 0.4% of the original helenalin was detected by liquid chromatography after the incubation for 3 days compared to that in Day 0 (Figure S1). Thus, we decided to treat RMS cells with helenalin only for 24 h in the study to find the mechanism of action, since over 95% of the compound persisted for 24 h. A longer time course might be complicated by the degradation of the compound. A previous study investigating the effect of helenalin on breast cancer (T47D) cells exhibited an IC_50_ of 1.3 µM after 72 h treatment [21], indicating that helenalin is less cytotoxic to the embryonic RMS cancer (RD) cells than breast cancer cells. We have also tested control fibroblast (non-tumour) cells, which showed IC_50_ of 9.26 μM after 24 h incubation, and 5.65 μM after 72 h incubation (Figure S2). Even though the IC50s of fibroblast cells are approximately 1.2–2.3-fold higher than those of RD and RH30 cells, helenalin could still induce adverse effects in patients if the drug was not targeted to the cancer cells.

### 2.2. Effect of Helenalin on Migration of RMS Cells

The cellular migration and invasion capabilities were evaluated by an in vitro wound healing assay. RMS cells were treated with drugs (DMSO (control), 2.5 µM helenalin, or 5 µM helenalin) for 24 h and a wound closure rate was evaluated using Equation (1) in Section 4.4.3 after the diameter of the wound was measured (Figure 1D,F). The wound closure rates (−2.7 ± 4.3% in RD cells, 18.7 ± 1.9% in RH30 cells) in RMS cells treated with 5 µM helenalin were significantly bigger than those treated with DMSO (57.8 ± 13.4% in RD cells, 48.2 ± 2.9% in RH30 cells). In RD cells, 24 h treatment of 5 µM helenalin actually increased the gap in the cell layer instead of narrowing (thus, the wound closure rate was the negative average value (−2.7%)), which implies that in vitro migration of RD cells might be completely hindered by helenalin treatment coupling with significant cellular death, while the regrowth in RH30 cells were limited a lesser degree than that of RD cells. This indicates that while the chemoresistance and migration ability of RH30 cells are both greater than those of RD cells, helenalin could still reduce in vitro cellular migration in both RMS cell lines (Figure 1E,G).

### 2.3. Assessment of Helenalin-Induced Cell Cycle Arrest and Cell Death

**Figure 2:**
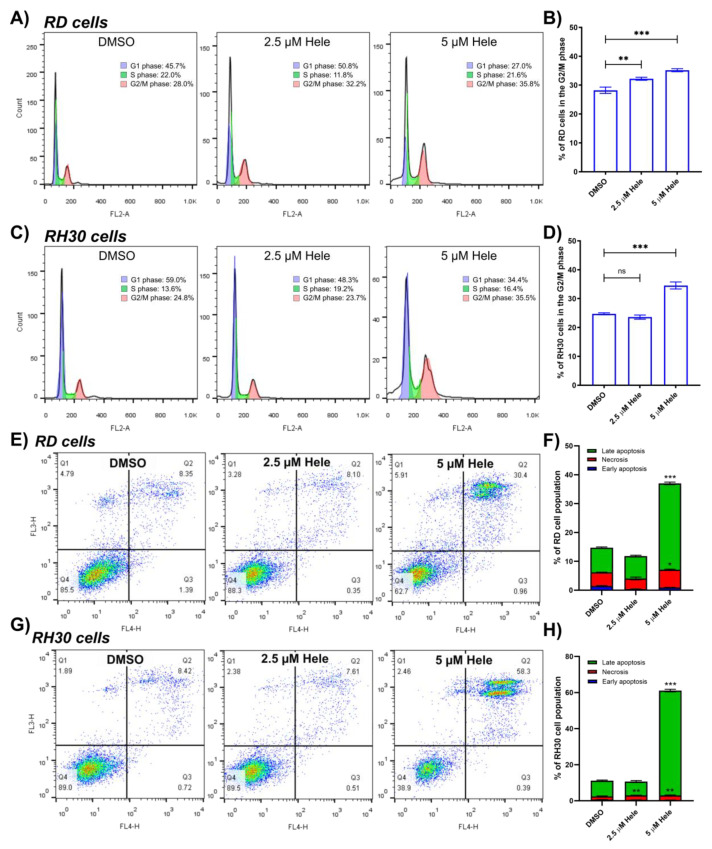
(**A**,**C**) The cell cycle analysis of RD and RH30 cells upon 24 h treatment of drugs by flow cytometry. (**B**,**D**) Comparative analysis of% of RD and RH30 cell population in the G2/M phase from the flow cytometric analysis. (**E**,**G**) The cell death analysis of RMS cells by flow cytometry after the PI and AV staining. (**F**,**H**) The percentages of early apoptotic (AV +/PI−), necrotic (AV−/PI+) and late apoptotic (AV +/PI+) cells upon 24 h treatment of drugs in RD and RH30 cells. The % of the early apoptotic, necrotic and late apoptotic cells treated with helenalin were compared with those treated with DMSO. Significances were tested using one-way ANOVA with Dunnett post hoc tests (ns *p* > 0.05, * *p* ≤ 0.05, ** *p* ≤ 0.01, *** *p* ≤ 0.001).

Cell cycle distribution of RMS cells was analysed by flow cytometry after helenalin treatment. Many mutagens, carcinogens and chemotherapeutic drugs generate extensive DNA damage during apoptosis or necrosis and the susceptibility of cellular DNA to damage by anti-tumour drugs can be evaluated through cell cycle analysis [22]. It was found that RMS cell populations in the G2/M phase increased significantly upon treatment with 5 µM helenalin compared to DMSO (negative control) treatment, while those at G0/G1 and S phases either showed no change or decreased. In RD cells, 2.5 µM helenalin increased the proportion of G2/M cells to 32.2 ± 0.5% and 5 µM helenalin to 35.2 ± 0.5% compared to 28.2 ± 1.1% in the control. RH30 cells exhibited an increase of the proportion of G2/M phase to 34.6 ± 1.2% upon 5 µM helenalin treatment compared to 24.7 ± 0.4% in the control (Figure 2A–D).

Helenalin-induced RMS cell death was characterised by the PI/annexin V (AV) staining assay. PI/AV staining is widely used to ascertain whether cells undergo apoptosis or necrosis through changes in cell membrane integrity and permeability [23]. PI is likely to penetrate the membranes of later apoptotic and necrotic cells, and stain double-stranded DNAs. Conversely, AV is likely to bind to phosphatidylserine, which might flip to the extracellular surface during apoptosis. Through flow cytometry, the PI−/AV+ (early apoptotic), PI +/AV+ (late apoptotic) and PI +/AV− (necrotic) populations in RMS cells treated with helenalin were analysed (Figure 2E,G). There was a significant increase in the population of necrotic and late apoptotic cells after 24 h treatment with helenalin. RD cells treated with 5 µM helenalin had 6.1 ± 0.2% necrotic cells and 29.9 ± 0.5% late apoptotic cells, compared to 4.7 ± 0.1% necrotic cells and 8.5 ± 0.3% late apoptotic cells in DMSO-treated groups (Figure 2F). RH30 cells comprised 2.6 ± 0.2% necrotic cells and 58.1 ± 0.2% late apoptotic cells after treatment with 5 µM helenalin compared to 1.8 ± 0.2% necrotic cells and 8.7 ± 0.4% late apoptotic cells in DMSO-treated groups (Figure 2H). However, there was no significant change in the number of early apoptotic RMS cells after helenalin treatment. This demonstrates that helenalin tends to trigger late apoptosis and/or necrosis in RD and RH30 cells. Since the necrosis is likely to be associated with reduced cancer survival and promoting tumour progression, the induction of apoptosis instead of necrosis is thought to be advantageous in cancer treatment [24,25,26]. The anti-tumour action of helenalin, by which the late apoptosis is a major mode in causing tumour cell death, might be beneficial for the treatment of tumours in clinical applications.

### 2.4. Evaluation of Action of Helenalin towards RMS Cell Death

#### 2.4.1. Oxidative Status

**Figure 3:**
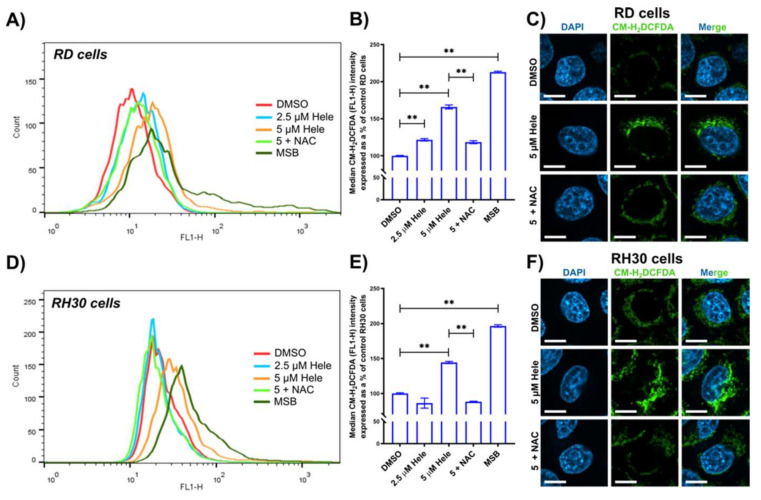
Oxidative stress levels in RMS cells. (**A**,**D**) Flow cytometric measurement of ROS levels indicated by CM-H_2_DCFDA (FL1-H fluorescence) in RD and RH30 cells. (**B**,**E**) Comparative analysis of ROS levels in RD and RH30 cells from flow cytometric measurements. (**C**,**F**) Confocal microscopy detection of DAPI and CM-H_2_DCFDA from RD and RH30 cells. Scale bars = 10 µm. Cells were treated with DMSO (0 µM helenalin, DMSO), 2.5 µM helenalin (2.5 µM Hele), 5 µM helenalin (5 µM Hele), 5 µM helenalin after pre-treatment of 5 mM N-acetyl-l-cysteine (NAC) for 2 h (5 + NAC) and 25 µM Menadione sodium bisulfite (MSB) for 24 h. Significances were tested using one-way ANOVA with Tukey post hoc tests (** *p* ≤ 0.01).

Flow cytometry with the 5-(and 6)-Chloromethyl-2′,7′-dichlorodihydrofluorescein diacetate, acetyl ester (CM-H_2_DCFDA) staining was used to investigate the levels of reactive oxygen species (ROS) in RMS cells. A positive control (25 µM menadione sodium bisulfite; MSB) was included in this analysis [27]. There was a significant increase in ROS (165.8 ± 2.7% in RD, 144.3 ± 1.6% in RH30) in the cells treated with 5 µM helenalin for 24 h compared to those treated with DMSO (negative controls) (Figure 3A,D). N-acetyl-l-cysteine (NAC) was used as an antagonist to helenalin in the oxidative stress pathway, since NAC has been reported to scavenge ROS and reduce the cellular oxidative stress [28]. NAC concentration was optimised to be the lowest concentration (5 mM) to induce the ROS scavenging activity in RMS cells without changing the cell viabilities (Figure S3) and the pre-treatment period was set to 2 h [28]. Two hours pre-treatment of 5 mM NAC followed by treatment with 5 µM helenalin resulted in a significant decrease in ROS of 47.5 ± 3.8% and 55.9 ± 2.2% in RD and RH30 cells, respectively (Figure 3B,E).

Qualitative images to indicate ROS were also taken by confocal microscopy using CM-H_2_DCFDA and DAPI (DNA indicator) (Figure 3C,F). When CM-H_2_DCFDA is oxidated by cellular ROS, the fluorescent adducts are released from the original molecules, leading to generation of green fluorescence inside cells [29]. Treatment with 5 µM helenalin for 24 h led to significantly higher fluorescence in both RD and RH30 cells compared to the controls, although to a lesser extent in RH30 cells. Pre-treatment with ROS antagonist NAC, however, quenched the ROS signals effectively in both RD and RH30 cells. This study indicates the pronounced ROS production by helenalin and the mitigation of helenalin-induced oxidative stress by NAC in RMS cells.

#### 2.4.2. Mitochondrial Response

**Figure 4:**
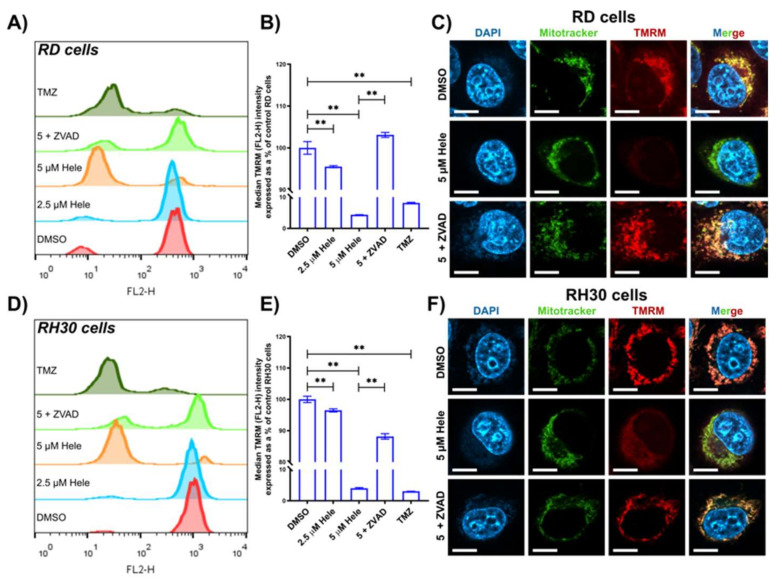
Mitochondrial responses in RMS cells. (**A**,**D**) Flow cytometric measurement of **Δ**𝚿m indicated by TMRM (FL2-H fluorescence) in RD and RH30 cells, respectively. (**B**,**E**) Comparative analysis of **Δ**𝚿m in RD and RH30 cells from the flow cytometric measurement,. (**C**,**F**) The confocal microscopy detection of DAPI, Mitotracker green and TMRM from RD and RH30 cells. The scale bars indicate 10 µm. Cells were treated with DMSO (0 µM helenalin, DMSO), 2.5 µM helenalin (2.5 µM Hele), 5 µM helenalin (5 µM Hele), 5 µM helenalin after pre-treatment of 100 µM ZVAD for 30 min (5 + ZVAD) and 400 µM Temozolomide (TMZ) for 24 h. Significances were tested using one-way ANOVA with Tukey post hoc tests (** *p* ≤ 0.01).

Mitochondrial membrane potential (**Δψ**m) in RMS cells was investigated after helenalin treatment, by flow cytometry with TMRM (**Δ**𝚿m indicator) staining. The positive control temozolomide (400 µM) was included in this investigation. Temozolomide is an alkylating agent used clinically for cancer therapy. It is known to induce lethal DNA damage followed by the caspase-dependent apoptosis, leading to mitochondrial depolarisation [30,31]. There was a considerable decrease of **Δ**𝚿m (4.4 ± 0.1% in RD & 4.0 ± 0.2% in RH30) in the cells treated with 5 µM helenalin for 24 h compared to those treated with DMSO (negative controls) (Figure 4A,D). Helenalin treatment (2.5 µM) also caused mitochondrial depolarisation, but was less severe than 5 µM helenalin treatment, signifying the dose-dependent **Δ**𝚿m disruption by the compound. Since the activation of caspases, e.g., caspase 3, correlates with initial mitochondrial membrane depolarisation, an inhibitor of pan-caspases was used. Therefore, the compound Z-VAD-FMK (ZVAD) which is thought to stabilise **Δ**𝚿m was used as an antagonist agent for the mitochondrial depolarisation pathway [32,33]. ZVAD concentration was optimised to be the minimal level required (100 µM) to induce the mitochondrial protection efficacy in RMS cells without affecting cell viability (Figure S4) and the pre-treatment period was set to 30 min [34]. The pre-treatment of cells with 100 µM ZVAD for 30 min followed by treatment with 5 µM helenalin led to a substantial increase of 98.7 ± 0.6% and 88.2 ± 0.7% in **Δ**𝚿m in RD and RH30 cells, respectively (Figure 4B,E).

Confocal microscopy was performed to observe the mitochondria of RMS cells using Mitotracker green (Mitochondrial localisation indicator), TMRM and DAPI (nuclear stain). TMRM is most likely to stain active mitochondria with intact **Δ**𝚿m, whereas Mitotracker green can accumulate live mitochondria regardless of **Δ**𝚿m [35]. The confocal imaging showed that 24 h treatment with 5 µM helenalin reduced TMRM fluorescence intensity in RMS cells, and also augmented Mitotracker green fluorescence intensity in RH30 cells (Figure 4C,F). The decrease of TMRM signals is correlated with a loss of **Δ**𝚿m induced by helenalin treatment, while the perinuclear clustering of mitochondria with a high Mitotracker green intensity is likely to stem from the increase of mitochondrial mass during the stress associated mitochondrial biogenesis as well as mitochondrial ROS production [36,37]. Pre-treatment of cells with ZVAD increased **Δ**𝚿m (that was decreased by helenalin alone) and prevented the clustering of mitochondria by decreasing the mitochondrial mass (that was increased by helenalin) in both RD and RH30 cells.

#### 2.4.3. Endoplasmic Reticulum (ER) Response

**Figure 5:**
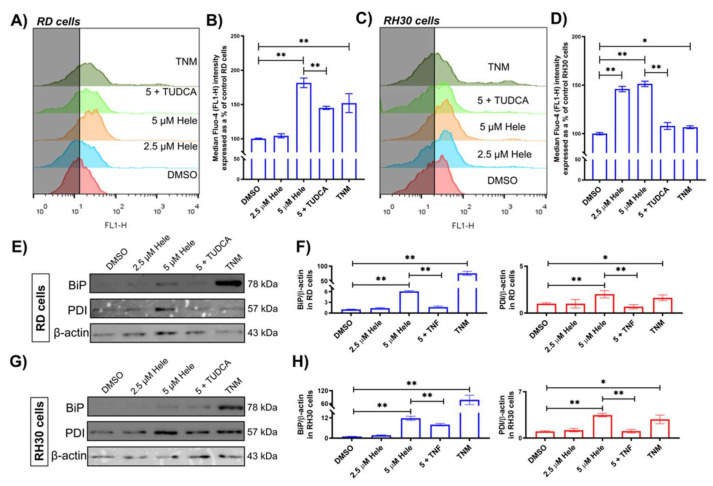
ER stress levels in RMS cells. (**A**,**C**) The flow cytometric measurement of intracellular Ca^2+^ indicated by Fluo-4 (FL1-H fluorescence) in RD and RH30 cells (The shadow is drawn up to the mean FL1-H value of the negative control (DMSO)). (**B**,**D**) Comparative analysis of Ca^2+^ levels in RD and RH30 cells from the flow cytometric measurement. (**E**,**G**) Immunoblotting results evaluating ER stress relating proteins (BiP & PDI) in RD and RH30 cells. (**F**,**H**) Comparative analysis of BiP & PDI protein levels in RD and RH30 cells by normalising the band intensities of BiP & PDI against β-actin using ImageJ. Cells were treated with DMSO (0 µM helenalin, DMSO), 2.5 µM helenalin (2.5 µM Hele), 5 µM helenalin (5 µM Hele), 5 µM helenalin after 24 h pre-treatment of 100 µM TUDCA (5 + TUDCA) and 10 µM tunicamycin (TNM, positive control) for 24 h. Significances were tested using one-way ANOVA with Tukey post hoc tests (* *p* ≤ 0.05, ** *p* ≤ 0.01).

The flow cytometric analysis with Fluo-4 (Ca^2+^ indicator) staining revealed that 24 h treatment of 5 µM helenalin increased the intracellular calcium levels to 181.6 ± 6.9% in RD (Figure 5A,B) and 151.5 ± 2.7% and RH30 cells (Figure 5C,D) compared to the cells treated with DMSO (negative controls). Tunicamycin (TNM, 10 µM) was included in this investigation as a positive control [38]. Tauroursodeoxycholic acid (TUDCA) was employed as an antagonist to helenalin because of its ability to attenuate ER stress by impeding unfolded protein response dysfunction [39]. The concentration of TUDCA was optimised to be the minimal level required (100 µM) to alleviate ER stress without affecting cell viability (Figure S5), and its pre-treatment period was set to 24 h [40]. As expected, 24 h pre-treatment with 100 µM TUDCA before helenalin addition reduced the calcium levels in RMS cells, which had been increased by helenalin treatment alone. The immunoblotting analysis indicated that helenalin treatment increased the levels of the ER stress associated proteins—binding immunoglobulin protein (BiP) and protein disulfide isomerase (PDI) in RMS cells (Figure 5E–H). BiP levels were increased by 6.1 ± 0.2-fold (in RD) and 11.8 ± 1.2-fold (in RH30), while PDI levels were increased 2.0 ± 0.4-fold (in RD) and 3.5 ± 0.3-fold (in RH30) in the cells treated with 5 µM helenalin for 24 h compared to the negative controls. TUDCA pre-treatment before helenalin addition reduced the levels of BiP and PDI, indicating that helenalin-induced ER stress in RMS cells might be mitigated by TUDCA.

#### 2.4.4. NF-**κ**B Activation

**Figure 6:**
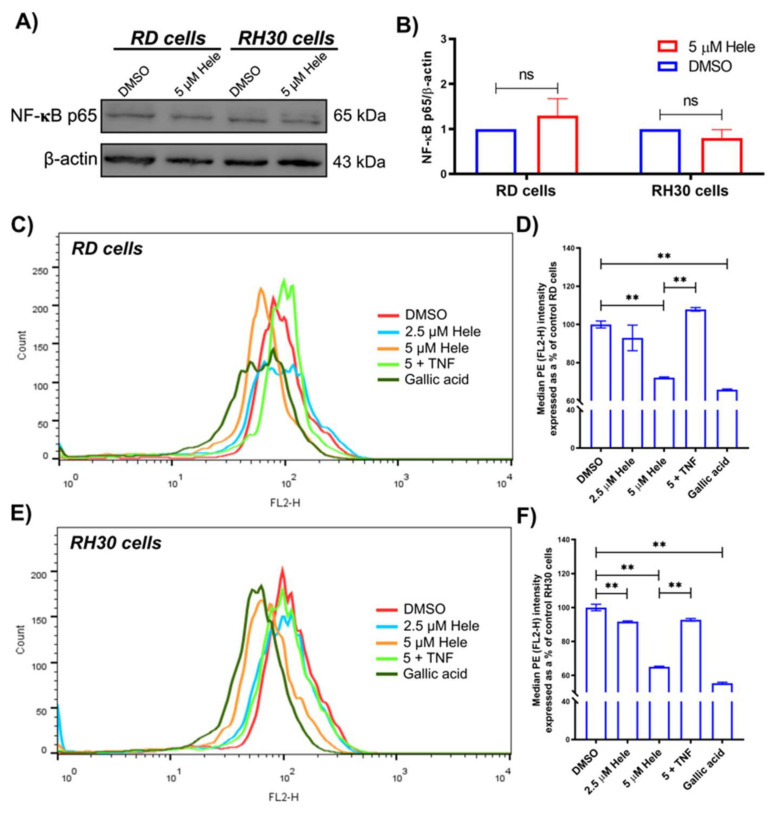
NF-**κ**B activation levels in RMS cells. (**A**) Immunoblotting result evaluating NF-**κ**B p65 expression levels using anti-NF-**κ**B p65 and anti-β-actin antibodies. (**B**) Comparison of NF-**κ**B p65 expression levels in RMS cells normalised against β-actin using ImageJ 1.53. (**C**,**E**) The flow cytometric measurement of NF-**κ**B activation levels indicated by anti-NF-**κ**B p65 pS529 antibody conjugated PE (FL2-H fluorescence) in RD and RH30 cells each. (**D**,**F**) The comparative analysis of NF-**κ**B activation levels from the flow cytometric measurement in RD and RH30 cells. Cells were treated with DMSO (0 µM helenalin, DMSO), 2.5 µM helenalin (2.5 µM Hele), 5 µM helenalin (5 µM Hele), 5 µM helenalin after pre-treatment with 5 ng/mL TNF-**α** for 1 h (5 + TNF) and 4 µg/mL gallic acid (positive control) for 24 h. Significances were tested using a two-tailed t-test (**B**) or one-way ANOVA with Tukey post hoc tests (**D**,**F**) (ns *p* > 0.05, ** *p* ≤ 0.01).

The impact of helenalin on the expression of NF-**κ**B p65 in RMS cells was assessed using immunoblotting. There were no differences detected in the NF-**κ**B p65 expression between the negative control (DMSO-treated cells) and 5 µM helenalin-treated cells in either RD or RH30 cells (Figure 6A,B). During NF-**κ**B activation, I**κ**Bα (inhibitor of nuclear factor kappa B) is ubiquitinated and degraded by IKKα/β (I**κ**B kinases), which leads to the phosphorylation of p65 for the translocation into the nucleus and the promotion of target gene transcription. NF-**κ**B p65 phosphorylation at serine 529 is associated with its transcriptional activities [41]. In the flow cytometric analysis using phosphorylated anti-NF-**κ**B p65 at Ser529 (p65 pS529), helenalin exhibited NF-**κ**B deactivation potency in RMS cells in the same manner as gallic acid (positive control) [42]. The levels of p65 pS529 in RD and RH30 cells were decreased to 72.1 ± 0.4% and 67.7 ± 0.3% of the negative controls after 24 h treatment with 5 µM helenalin (Figure 6C,E). Tumour necrosis factor alpha (TNF-**α**) is known to induce the canonical NF-**κ**B activation upon binding to TNFR (TNF-**α** receptors) and so it was used as an antagonist in the NF-**κ**B activation pathway [43]. TNF-**α** concentration was optimised to be the minimum (5 ng/mL) to increase NF-**κ**B levels without affecting cellular viability (Figure S6) and the pre-treatment period was set as 1 h [7]. Pre-treatment of TNF-**α** for 1 h before helenalin treatment, increased NF-**κ**B activation level back to the normal levels (107.9 ± 0.9% in RD cells, 96.5 ± 0.8% in RH30 cells) (Figure 6D,F).

#### 2.4.5. Cell Survival Improvement by Different Antagonists

**Figure 7:**
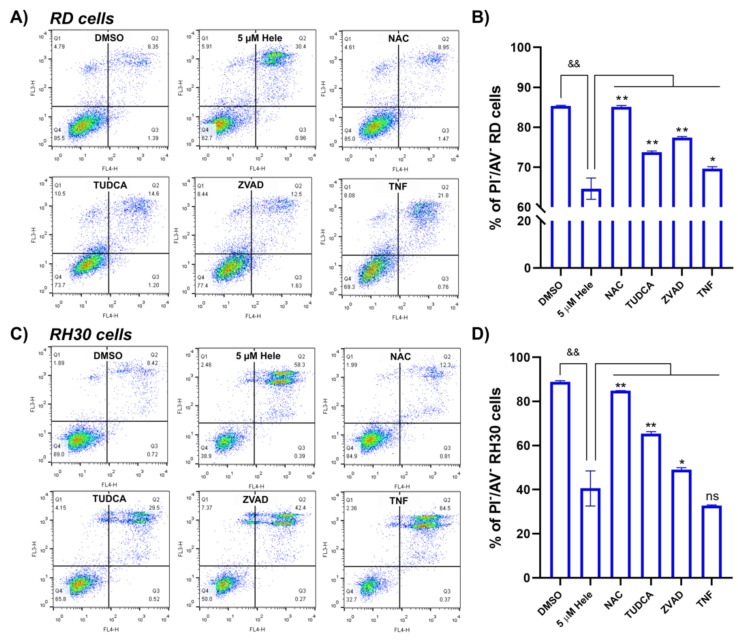
Cell survival assessment of RMS cells using PI/AV staining. (**A**,**C**) The flow cytometric measurement of cell viabilities of RD and RH30 cells stained by PI and AV each. (**B**,**D**) Comparison of % of PI−/AV− (live) cell populations in RD and RH30 cells each. Cells were treated with DMSO (0 µM helenalin, DMSO) and 5 µM helenalin for 24 h after pre-treatment of DMSO for 24 h (5 µM Hele), 5 mM NAC for 2 h (NAC), 100 µM TUDCA for 24 h (TUDCA), 100 µM ZVAD for 30 min (ZVAD) and 5 ng/mL TNF-**α** for 1 h (TNF). Significances were tested using one-way ANOVA with Tukey post hoc tests (ns *p* > 0.05, * *p* ≤ 0.05, ** (&&) *p* ≤ 0.01).

Thus far, we have demonstrated that helenalin induces oxidative stress, mitochondrial depolarisation, ER stress and NF-**κ**B deactivation. These are considered to be the major pathways in triggering the anti-tumour activity in RMS cells. To establish the mechanism of action of helenalin in RMS cells it is necessary to delineate the dominant pathways, and the order in which signalling molecules are produced. We have shown that many antagonistic chemicals were able to reverse the action of helenalin in the individual pathways, i.e., NAC in ROS generation, ZVAD in mitochondria dissipation, TUDCA in ER stress and TNF-**α** in the NF-**κ**B deactivation pathways. Based on these results, the effect of the antagonists on survival in helenalin-induced RMS cell death were compared. RMS cells were pre-treated with antagonists followed by helenalin treatment and subjected to the PI/AV staining assay followed by flow cytometry (Figure 7A,C). The pre-treatments with one or two antagonist(s) were shown to promote survival from helenalin-induced RMS cell death. Among the antagonist pre-treatments, NAC exhibited by far the highest survival improvement from cell death, such that the proportion of AV^−^/PI^−^ (live) RD and RH30 cells could be increased to 85.1 ± 0.4% and 84.8 ± 0.1% from 64.6 ± 2.6% and 42.2 ± 6.2%, respectively, after NAC pre-treatment compared to those treated with helenalin alone. Pre-treatment with ZVAD or TUDCA has also resulted in a significant increase of 12.8 ± 2.7% (ZVAD) and 23.1 ± 5.9% (TUDCA) in the survivals of RD and RH30 cells compared to those without antagonists, although neither ZVAD nor TUDCA showed cell survival recovery as high as that seen with NAC. This outcome demonstrates that ROS generation might be the primary pathway in the heleanlin-induced RMS cell death. We have further investigated three inessential pathways, i.e., mitochondrial depolarisation, ER stress and NF-**κ**B deactivation, to find out which one would support the oxidative stress mediated cell death the most. The antagonists of inessential pathways (ZVAD, TUDCA and TNF-**α**) were treated into RMS cells in addition to NAC (an antagonist for the main cell death causing pathway oxidative stress) followed by the PI/AV assay. Various combinations of ZVAD, TUDCA and TNF-**α** were also assessed. The combination of NAC with either TNF-**α**, ZVAD, or TUDCA did not increase the rescue of cells compared to pre-incubation with NAC alone (Figure S7A–D). These dual experiments reinforce the idea that oxidative stress is the primary mechanism, while the single compound experiments indicate that there could be a contribution to cell death via the other pathways.

**Figure 8:**
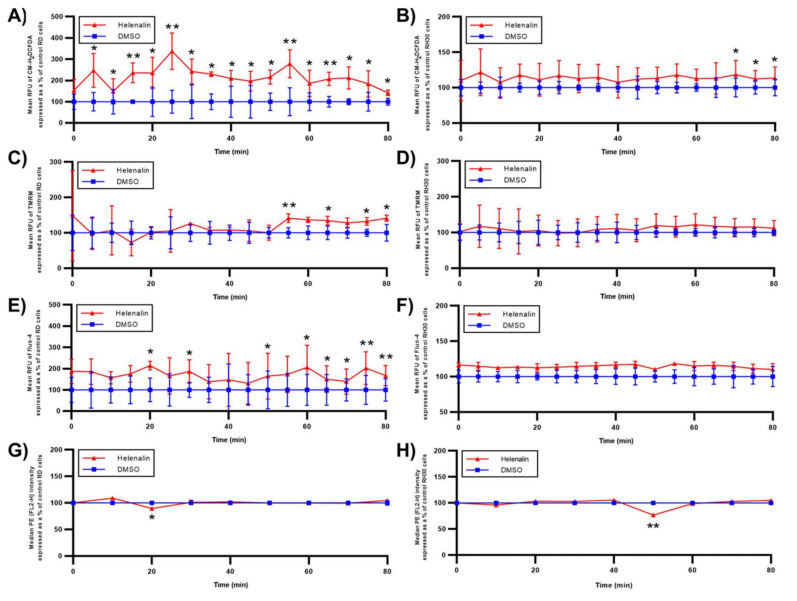
Signal changes from several pathways by helenalin treatment in RD and RH30 cells. (**A**,**B**) ROS levels (mean RFU of CM-H_2_DCFDA) of RD and RH30 cells, respectively, treated with DMSO and 5 μM helenalin. (**C**,**D**) MMP levels (mean RFU of TMRM) of RD and RH30 cells, respectively, treated with DMSO and 5 μM helenalin. (**E**,**F**) Ca^2+^ levels (mean RFU of Fluo-4) of RD and RH30 cells, respectively, treated with DMSO and 5 μM helenalin. (**G**,**H**) NF-**κ**B p65 506 phosphorylation levels (mean RFU of PE) of RD and RH30, respectively, cells treated with DMSO and 5 μM helenalin. All treatments were for 80 min. Significances were tested using a two-tailed t-test (* *p* ≤ 0.05, ** *p* ≤ 0.01).

To find the mechanism by which helenalin affects RMS cells the order in which events occur was investigated. As such, the levels of ROS, **Δ**𝚿m, Ca^2+^ and NF-**κ**B p65 pS529 in RMS cells over 80 min immediately after helenalin treatment were determined (Figure 8). In RD cells (Figure 8A,C,E,G), ROS levels began increasing from 5 min after helenalin addition. Subsequently Ca^2+^ and NF-**κ**B p65 phosphorylation levels started to change after 20 min, while there was an increase in **Δ**𝚿m levels (hyperpolarisation) at 55 min. The mitochondrial hyperpolarisation, which is believed to be associated with high ROS levels in the cytosol, could lead to subsequent mitochondrial dissipation [44]. Conversely, RH30 cells (Figure 8B,D,F,H) exhibited the one-point change of NF-**κ**B activation at 50 min and the steady increase of ROS levels from 70 min, while **Δ**𝚿m and Ca^2+^ levels remained unchanged over 80 min. This result supports our speculation that while oxidative stress is the main pathway induced by helenalin, RMS cell death might also plausibly be supported by NF-**κ**B deactivation.

## 3. Discussion

The sesquiterpene lactone, helenalin, has been studied herein, as a candidate chemotherapeutic material, due to its reported ability to impede the NF-**κ**B signalling pathway, which is associated with anti-apoptosis, metastasis and chemoresistance of tumour cells [7,13]. RMS is a paediatric sarcoma possessing genetic aberrations that cause both eRMS and aRMS cells to circumvent apoptosis or necrosis induced by chemotherapy and radiotherapy. Many chemotherapy regimens such as VAC (vincristine, actinomycin D and cyclophosphamide) and IVA (ifosfamide, vincristine and actinomycin D) procedures have been implemented for RMS treatment, but the effectiveness in those with recurrent or metastatic RMS remains insufficient [45]. It necessitates the development of new therapeutic agents that can combat the survival machinery of malignant cells, and helenalin is considered as one of them. A number of studies that have been performed to screen for anti-tumour efficacy of helenalin on various adult tumour cell lines suggest that helenalin-induced cell death occurs concomitantly with mitochondrial membrane permeabilisation, ER stress accompanied by calcium discharge into cytosol, ROS generation and NF-**κ**B deactivation [16,17,19,46]. This study was focused on the elucidation of the fundamental pathway involved in helenalin-induced RMS cell death. This was achieved by comparing the contribution of several mechanistic routines, such as oxidative stress, mitochondrial dissipation, ER stress and NF-**κ**B inhibition, to the cellular death using antagonistic materials.

In this study, we have found that the IC_50_ of helenalin against both RD and RH30 cells for 24 and 72 h lies at a single-digit-micromolar range, which indicates a high cytotoxicity in vitro. Vincristine, which is one of the anti-tumour drugs of VAC regimen used in the clinical area, was reported to have a IC_50_ of 1.97 nM and 1.03 nM against RD and RH30 cells for 72 h in vitro each [47], which signifies that vincristine is about 2500 times more toxic than helenalin. Furthermore, the IC_50_ of vincristine for fibroblast cells is 96 nM, which is almost 100 times that of RD and RH30 cells, while helenalin exhibited about 2.3-fold difference in the IC_50_s between the normal and RMS cells [48]. The narrow therapeutic window of helenalin may have restricted clinical use to date; however, targeted nanoparticles which encapsulate helenalin could both improve the therapeutic index and lessen side effects. As cell migration is an indispensable part of metastasis required during every phase of the metastatic cascade, in vitro migration potential of RMS cells was investigated under helenalin treatment. In both RD and RH30 cells, the wound closure rate was decreased with the elevation of helenalin concentration, suggesting that helenalin suppresses the metastatic capacity of RMS cells in vitro. The gap distance in RD cells treated with 5 µM helenalin, which had got bigger than that 24 h earlier, is associated with the significant cytotoxicity as well as the inhibition of migration caused by the high concentration of helenalin. It is of particular interest that very low concentrations (0.02 & 0.2 µM) of helenalin are able to advocate cellular migration of HaCaT keratinocytes instead of suppressing in vitro movement [49]. This result indicates that helenalin might act very differently according to its concentration, e.g., between a concentration lower than 0.2 µM and higher than 2 µM, but this needs further investigation. 

Cells are most likely to undergo a cell cycle arrest at certain checkpoints in response to the activation of pathways leading to programmed cell death. If DNA damage was exposed at the checkpoints, cells would not be able to initiate mitosis until the errors were repaired, and any irreparable damage could lead to apoptosis [50]. We found that helenalin caused cell cycle arrest in the G2/M phase in RMS cells. G2 and M phases are the stages where cells prepare for mitosis after DNA replication and undergo cell division, and the G2 phase checkpoint ensures DNA integrity within the chromosomes [51]. Thus, the helenalin-induced G2/M phase arrest is associated with the complications of DNA replication in RMS cells, which might be one of the mechanisms of helenalin in causing tumour cell death. Apoptosis is the most common cell death regulated by host cells, which consequently leads to morphological changes such as cell shrinkage, membrane blebbing and chromatin fragmentation, while necrosis typically takes place as an accidental form of cell death, resulting in organelle breakdown, cytoplasm vacuolation, membrane breakdown, which can trigger immune responses subsequently [52,53]. In a previous study, Lim et al. reported that helenalin was able to increase the levels of cleaved caspase 3, caspase 9 and PARP in a dose- and time-dependent manner in ovarian, colon and breast cancer cells. This indicated that helenalin would induce apoptosis in tumour cells [16]. Our results suggested that helenalin is highly likely to induce late apoptosis in both RD and RH30 cells, whereas necrotic cell death is induced at low rates (6.1 ± 0.2% in RD cells, 2.6 ± 0.2% in RH30 cells). Necrosis generally takes place as an alternative cell death process to apoptosis or autophagy in tumour cells that are resistant to conventional cell death such as apoptosis [54]. Helenalin has been reported to cause apoptosis in various cells including renal carcinoma cells [17], CD4+ T cells [55] and leukaemia cells [19], and to trigger necrosis in apoptosis-resistant cells such as Bcl-2 overexpressing leukaemia cells [46]. Taking the previous studies into consideration, it implies that RMS cells which tend to undergo apoptosis after helenalin treatment have relatively high sensitivity to undergo programmed cell death.

Cellular oxidative stress is associated with ROS generation and reduced glutathione (GSH) depletion, which can instigate apoptotic or necrotic cell death [56,57]. Since the functional moieties of helenalin such as α-methylene-ɣ-lactone and cyclopentenone can react with bio-nucleophiles, especially the thiol groups of cysteine residues in various proteins including GSH, it can increase the cellular ROS levels, which is thought to be one of the main actions in killing tumour cells. We found that ROS levels in RMS cells increased after helenalin treatment in a dose-dependent manner, indicating that helenalin could sabotage the antioxidant enzyme system in RMS cells. Oxidative stress triggered by helenalin was also reported in previous studies [8,58]. NAC, which is a known ROS scavenger, antagonised the helenalin-induced ROS generation in both RD and RH30 cells and consequently reduced ROS levels.

Mitochondria are known to trigger caspase-dependent or independent apoptosis through mitochondrial outer membrane permeabilisation (MOMP). They can also induce necrotic cell death in response to permeabilisation of both the inner and outer membrane triggered by calcium overload or oxidative stress [59]. Our investigation revealed that helenalin decreased **Δ**𝚿m of mitochondria while augmenting the mitochondrial mass and/or the mitochondrial ROS. ZVAD, a pan-caspase inhibitor, has been outlined to inhibit mitochondria-mediated apoptosis mainly by protecting **Δ**𝚿m of mitochondria [33]. It was therefore used as an antagonist to helenalin in the mitochondrial depolarisation pathway. The pre-treatment of cells with ZVAD reduced mitochondrial dissipation by increasing **Δ**𝚿m back to the normal state. Isoalantolactone, another sesquiterpene lactone, was able to suppress the ratio of Bcl-2 to Bax (apoptosis-related regulator) proteins that result in caspase cleavage for the mitochondrial dissipation [60]. Mitochondria-mediated apoptosis has been reported to be induced by several sesquiterpene lactones including britannin [61] and isocostunolide [62]. Even though the alkylating groups of sesquiterpene lactones are associated with the permeabilisation and dissipation of **Δ**𝚿m, it is still not clear whether or not the mitochondrial impairment is directly induced through the inhibition of Bcl-2 family by sesquiterpene lactones.

The ER is the organelle responsible for the regulation of Ca^2+^ storage and release, as well as environmental, physiological and pathological abuses. Nutritional imbalances could activate the unfolded protein response (UPR) which results in ER stress. During ER stress, significant amounts of Ca^2+^ are released into the cytosol, triggering apoptosis or autophagy, and the levels of chaperone proteins such as BiP and protein disulfide isomerase (PDI) increase to assist misfolded proteins to refold properly [63,64]. We found that intracellular Ca^2+^ levels and the levels of BiP and PDI proteins in RMS cells were increased by helenalin treatment. It is widely accepted that ER stress is likely to cause autophagy by enforcing the dismantled ER to be engulfed by autophagosomes, and restoring ER homeostasis [65]. Lim et al. claimed that helenalin increased autophagic cell death markers (Atg12 & LC3-B) in ovarian cancer cells, which induced cell death via apoptosis or autophagy [16]. ER stress caused by helenalin treatment in RMS cells in this study is in agreement with the idea that helenalin might induce autophagy. The restoration of calcium levels and protein markers related to ER stress upon TUDCA pre-treatment suggests that TUDCA plays an antagonistic role against helenalin in the ER stress pathway, which is a consistent result with previous studies [39,66].

Helenalin has been reported to suppress the expression of NF-**κ**B p65 in ovarian cancer cells [16] or abrogate NF-**κ**B signalling by inhibiting DNA binding activity of p65 in several cell lines including leukemia Jurkat (J16) cells [46] and hepatic stellate (HSC-T6) cells [67]. We observed that helenalin decreased the phosphorylation levels of NF-**κ**B p65 at Serine 529, although it had no significant effect on the expression of NF-**κ**B p65. Since the phosphorylation of Serine 529 affects transcriptional activity of the p65 enzyme, it can be deduced that helenalin downregulates NF-**κ**B activation through interference with the transcriptional activities of its target genes [41]. TNF-**α** is the most potent physiological inducer of NF-**κ**B, since the binding of TNF-**α** to TNF receptor 1 (TNFR1) triggers the subsequent recruitment of TRADD, TRAF, cIAP and RIP1, which mediates the proximity of TAK to IKK complex and induces the nuclear translocation of NF-**κ**B p65/p50 dimers [43]. Indeed, pre-treatment of RMS cells with TNF-**α** restored the NF-**κ**B activation levels lowered by helenalin treatment back to the control levels. It has been reported that two free cysteine residues around the DNA binding loop of the p65 enzyme are prone to alkylation, which leads to the suppression of its association with DNA binding regions. Since helenalin has two alkylating groups, they might mediate the crosslink between two cysteine residues of p65 to inhibit DNA binding [68]. Other sesquiterpene lactones with only one alkylating group showed lower inhibitory effect on p65 activity, mainly because the hindrance of the DNA binding of one p65 enzyme entails at least two molecules to alkylate two residues [69].

**Figure 9:**
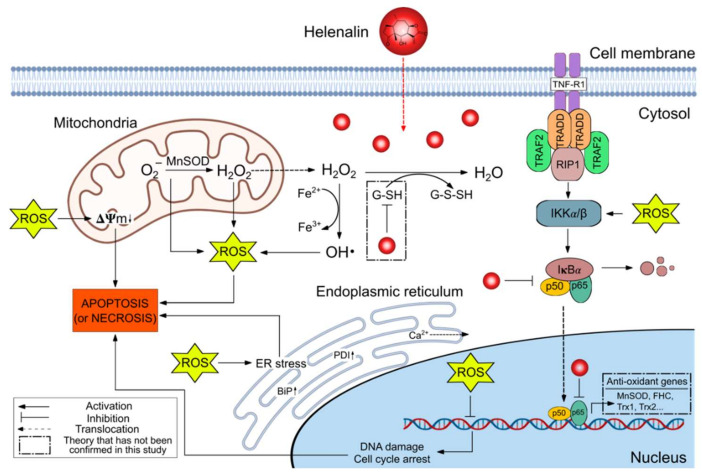
Proposed mechanism of helenalin in triggering RMS cell death. MnSOD: Manganese superoxide dismutase; ROS: Reactive oxygen species; **Δ**𝚿m: Mitochondrial membrane potential; G-SH: Glutathione in the reduced form; G-S-S-H: Glutathione in the disulfide form; TRADD: Tumour necrosis factor receptor type 1- associated DEATH domain protein; RIP1: Receptor interacting protein 1; TRAF 1 & 2: Tumour necrosis factor receptor- associated factor 1 & 2; IKK α/β: I**κ**Bα kinase α/β; I**κ**Bα: Inhibitor of nuclear factor kappa B; p65/p50: NF-**κ**B enzyme dimer; FHC: Ferritin heavy chain protein; Trx1 & 2: Thioredoxin-1 & 2 proteins.

To elucidate the mechanism of action of helenalin in RMS cells, we have applied the antagonism principle to recognise the dominant action of helenalin in causing cell death. We have chosen specific and distinctive antagonists (NAC, ZVAD, TUDCA, TNF-**α**) for the most widely published pathways (oxidative stress, mitochondrial dissipation, ER stress and NF-**κ**B deactivation, each) and then demonstrated that the pre-incubation of antagonists prior to helenalin treatment alleviated the stress from their corresponding pathways in RMS cells. The survival-improvement-by-antagonists experiment was performed by evaluating the viabilities of RMS cells pre-treated with the antagonists followed by helenalin treatment. Among solo-antagonist pre-treatments, NAC, which is an antagonist for the oxidative stress pathway, improved RMS cell viability the most, and ZVAD and TUDCA also significantly enhanced viabilities of RD and RH30 cells, respectively. Morrison et al. suggested that different cell types would take different routes of cell death in response to a chemotherapeutic natural product, Ophiobolin A [35]. This might explain the reason why RD and RH30 cells have different antagonists (ZVAD & TUDCA, respectively) to ameliorate their survivals besides NAC and that’s why researchers have perceived the different pathways susceptible to helenalin in different cancer cell lines up to now. Our outcome illustrates that the oxidative stress pathway might be the main action of helenalin in inducing RMS cell death. In the investigation to find out whether the oxidative stress-mediated cell death also involved other pathways, it was found that the pre-treatment with either TNF-**α**, ZVAD or TUDCA did not increase the cell rescue above that seen with NAC alone. This result reinforces the fact that helenalin-induced RMS cell death mostly occurs through oxidative stress, with possible small-scale contributions to cell death from mitochondrial depolarisation, ER stress and NF-**κ**B inhibition (Figure 9).

In our proposed mechanism, α-methylene-ɣ-lactone groups of helenalin would alkylate thiol groups of antioxidant molecules, (e.g., glutathione), and antioxidant enzymes, (e.g., glutathione reductase), to destroy the redox balance, leading to intracellular ROS generation [11]. ROS usually activates the IKK complex to degrade I**κ**B from NF-**κ**B p65/p50 complex, and the translocated NF-**κ**B dimers enhance the expression of antioxidant proteins such as manganese superoxide dismutase (MnSOD), Ferritin Heavy Chain (FHC) and Thioredoxin-1 & 2 (Trx1 & 2), leading to diminution of the oxidative stress [70]. The negative feedback of the NF-**κ**B signalling pathway on the cellular oxidative status can be blocked by helenalin, ensuring the undisturbed oxidative stress towards RMS cells. Moreover, ROS molecules (or helenalin itself) permeabilise the mitochondrial membrane and induce ER stress which results in the release of Ca^2+^ from ER and increases ER stress-related proteins such as BiP and PDI. In view of the fact that ZVAD, a pan-caspase inhibitor, showed lower survival potency than NAC (a ROS scavenger), helenalin-induced RMS cell death might undergo caspase-independent apoptosis or necrosis rather than caspase-dependent apoptosis. The in vitro mechanism of action of helenalin was investigated in this study; it would be beneficial to carry out a further evaluation on the anti-tumour efficacy and side effects of the compound on RMS tumour-bearing animals. This could provide further insights into its potential for clinical application. Most crucially, such in vivo studies need to address the stability of helenalin under the physiological conditions and the therapeutic window.

## 4. Materials and Methods

### 4.1. Materials

Two RMS cell lines, RD (ATCC no. CRL-7763) and RH30 (ATCC no. CRL-2061), were used in this study. RD cells originated from a tumour in the muscle of a 7-year-old female, and RD cells were from an alveolus tumour of a 17-year-old male. Helenalin was purchased from Abcam, Cambridge, UK. L-glutamine, penicillin/streptomycin, 5-(and 6)-Chloromethyl-2′,7′-dichlorodihydrofluorescein diacetate, acetyl ester (CM-H_2_DCFDA), Mitotracker green FM, tetramethyl rhodamine (TMRM) and fluo-4 were purchased from Life Technologies, Inchinnan, UK. Dulbecco’s modified Eagle’s medium (DMEM), fetal bovine serum (FBS), Dulbecco’s Phosphate Buffered Saline (DPBS), RNase A, Triton-X, 4% paraformaldehyde and MTT (3-(4,5-dimethylthiazol-2-yl)-2,5-diphenyltetrazolium bromide) were obtained from Sigma-Aldrich, Gillingham, UK. For the cell cycle and death analysis, we purchased propidium iodide staining solution (Cambridge Bioscience, Cambridge, UK), annexin V binding buffer (BioLegend, London, UK) and allophycocyanin conjugated annexin V (APC AV; BioLegend, London, UK). We used the antagonistic chemicals such as N-acetyl-L-cysteine (Sigma-Aldrich, Gillingham, UK), Z-VAD-FMK (Stratech Scientific, Cambridge, UK), tauroursodeoxycholic acid (Cambridge Bioscience, Cambridge, UK) and human tumour necrosis factor (TNF)-alpha protein (Bio-Techne, Abingdon, UK). The positive control compounds such as menadione sodium bisulfite (Sigma-Aldrich, Gillingham, UK), temozolomide (Stratech Scientific, Cambridge, UK), tunicamycin (Cambridge Bioscience, Cambridge, UK) and gallic acid (VWR International, Lutterworth, UK) were also included in this study. For the immunoblotting assay, RIPA lysis buffer system (Insight Biotechnology, Wembley, UK), Novex Tris-Glycine Mini Gels (Life Technologies, Inchinnan, UK), ECL western blotting substrate (Promega, Chilworth Southampton, UK), anti-β-actin antibody (Bio-Techne, Abingdon, UK), anti-NF-**κ**B antibody pS529 (Miltenyi Biotec, Surrey, UK) and ER stress antibody sampler kit including anti-BiP and anti-PDI antibodies (Cell Signaling Technology, London, UK) were purchased.

### 4.2. Cell Culture and Cell Viability Assay

Cells were grown in DMEM supplemented with 10% FBS, 2 mM L-glutamine and 100 U/mL penicillin/0.1 mg/mL streptomycin and incubated at 37 °C in a 5% CO_2_ atmosphere. Cell proliferation was evaluated using the MTT assay [71].

### 4.3. In Vitro Wound Healing Assay

For in vitro wound healing assay, cells were seeded in 24-well plates at a density of 2 × 10^5^ cells/well and incubated to confluence. Then, an artificial wound was created using a P200 pipette tip. Then cells were washed twice with PBS and incubated with drugs for 24 h. The distance between the sides of the scratch was measured under a Motic AE31 microscope and the wound closure rates were estimated using Equation (1) (GD_0 h_: gap distance at 0 h after drug treatment, GD_24 h_: gap distance at 24 h after drug treatment).

(1) $$\mathrm{Wound}\;\mathrm{Closure}\;\mathrm{Rate}\;\left(\%\right)=\frac{{\mathrm{GD}}_{0\;h}-{\mathrm{GD}}_{24\;h}}{{\mathrm{GD}}_{0\;h}}\times 100$$

### 4.4. Flow Cytometry

#### 4.4.1. Cell Cycle Analysis

Cells in the different cell cycle stages have various amounts of DNA, i.e., the cells might possess 2 sets, 2 to 4 sets or 4 sets of chromosomes (2n, 2 to 4n, 4n) in the G1, S and G2/M phases, respectively. In this investigation, we permeabilised RMS cells using ethanol, stained chromosomes with propidium iodide (PI) and estimated the amount of DNA by measuring PI fluorescence intensities, since the number of chromosomes is proportional to the amount of PI. Cells were seeded in 6-well plates at 5 × 10^5^ cells/well and allowed to adhere overnight. Cells were treated with drugs for 24 h and then washed with PBS and trypsinised. The non-adherent cells (from the washing step) and adherent cells (from the trypsinisation step) are collected and centrifuged at 500× *g* for 5 min and washed with PBS (Splitting step). The cells were fixed in 500 µL of 70% ethanol for 30 min on ice and resuspended with 500 µL of PBS containing 40 µg/mL propidium iodide staining solution (PI), 0.1 mg/mL RNase A and 0.1% Triton X-100 followed by incubation at room temperature (RT) for 30 min. Finally, the cells were subjected to flow cytometry analysis using a BD FACSCalibur flow cytometer by evaluating cells using a 488 nm laser for excitation and a bandpass filter at 585/42 nm for emission (FL2-A channel).

#### 4.4.2. Evaluation of Cell Death Status

Cells were washed with 200 µL of cold Annexin V binding buffer twice, after the splitting step (described in ***cell cycle analysis***) and incubated with 50 µL of Annexin V binding buffer containing 5 µL of PI and 2.5 µL of APC AV at 37 °C for 15 min. Then, 450 µL of AV binding buffer was used to resuspend cells prior to the analysis. Lastly, the cells were subjected to flow cytometry analysis by evaluating cell population in both FL3-H (a 488 nm laser for excitation and a long pass filter at 670 nm for emission) and FL-4-H channel (a 633 nm laser for excitation and a bandpass filter at 661/16 nm for emission).

#### 4.4.3. ROS and Mitochondrial Membrane Potential (MMP) Analysis

Cells were incubated with pre-warmed phenol free DMEM containing 2 µM CM-H_2_DCFDA or 500 nM TMRM at 37 °C for 30 min, after the splitting step (described in Section 4.4.1). The stained cells were washed with PBS twice and 500 µL of cell suspension were subjected to flow cytometric analysis by evaluating them in the FL1-H (a 488 nm laser for excitation and a bandpass filter at 530/30 nm for emission) or FL2-H channel (a 488 nm laser for excitation and a bandpass filter at 585/42 nm for emission) for the ROS and MMP analysis, respectively.

#### 4.4.4. Detection of Intracellular Antibody Binding

Cells were fixed by incubating cells with 4% paraformaldehyde for 10 min at RT, followed by washing with PBS, after the splitting step (described in Section 4.4.1). Cell permeabilisation was performed by incubating cells with 0.1% Triton X-100 in PBS for 10 min at RT, followed by washing with PBS twice. Cells were resuspended in 50 µL of 0.1% Triton X-100 in PBS and treated with 5 µL of anti-NF-**κ**B antibody pS529 conjugated with phycoerythrin (PE) for 30 min in the dark condition at RT. After washing cells with PBS twice, the fluorescence intensity of PE was quantified using a 488 nm laser for excitation and a bandpass filter at 585/42 nm for emission (FL2-H channel) by flow cytometry [72].

### 4.5. Immunoblotting

Cell lysates were prepared using RIPA lysis buffer system and protein concentration was determined using the BCA assay. Subsequently, 30 µg of total proteins were denatured and subjected to SDS-PAGE at 180 V for 1 h, followed by a transfer to PVDF membrane at 25 V for 9 min. The membranes were blocked with 5% milk in Tris-buffered Saline with 0.1% Tween-20 (TBST) at RT for 1 h and probed with anti-BiP (1:1000 dilution), anti-PDI (1:1000 dilution) and anti-β-actin (1:200 dilution) monoclonal antibodies as primary antibodies at 4 °C overnight. After washing three times in TBST, the membranes were incubated with horseradish peroxidase-conjugated secondary antibodies at RT for 1 h. The blots were revealed by ECL western blotting substrate and visualised in G:Box F3 (Syngene).

### 4.6. Confocal Microscopy

Cells were seeded in 6-well plates at 5 × 10^5^ cells/well and allowed to adhere overnight. Then the cells were treated with drugs for 24 h and were trypsinised, followed by incubation with CM-H_2_DCFDA (for ROS detection), Mitotracker green (for mitochondria localisation detection) and TMRM (for MMP detection) for a designated time according to their protocols. Cells were fixed in 4% paraformaldehyde for 10 min and transferred into slides through spinning them at 800 rpm for 3 min on cytospin before DAPI staining with the nucleus. Confocal microscopy images were acquired using UltraView spinning disk system (PerkinElmer) comprising CSU-X1 spinning disk head (Yokogawa) and Volocity software.

### 4.7. Statistical Analysis

All data were presented as Mean ± Standard Deviation (SD) and plotted using GraphPad Prism 8.0.2. Statistical analyses were performed by two-tailed paired Student’s *t*-test, one-way ANOVA with Dunnett post hoc tests and one-way ANOVA with Tukey post hoc tests in Microsoft Excel and GraphPad Prism 8.0.2. *p* < 0.05 was taken as the criteria for statistical significance. All experiments were performed in triplicates and repeated on at least two separate occasions.

## 5. Conclusions

Taken together, it has been found that helenalin induces a serious cytotoxicity towards eRMS (RD) cells and aRMS (RH30) cells in a dose- or time-dependent manner, or both. In vitro potency of helenalin was exerted through triggering G2/M cycle arrest and promoting late apoptosis in RMS cells. The most widely published pathways of helenalin in causing tumour cell death i.e., oxidative stress, mitochondrial depolarisation, ER stress and NF-**κ**B inhibition pathways, were included in the mechanism study. We have perceived that pre-treatment of cells with antagonists, i.e., NAC, ZVAD, TUDCA and TNF-**α**, attenuated the degree of stress from the corresponding pathways induced by helenalin. The survival-improvement-by-antagonists experiment demonstrated that the combined inhibition of both oxidative stress and NF-**κ**B activation by their antagonists led to the highest survival improvement from helenalin-induced RMS cell death. Thus, we propose that helenalin is likely to alkylate antioxidant molecules for ROS generation and it might also suppress the DNA binding activities of NF-**κ**B p65 to downregulate the expression of antioxidant protein, so that the cell survival mechanisms of RMS cells to prevent apoptosis or necrosis might be sabotaged effectively. The oxidative stress resulting from the ROS generation is likely to be the main mechanism by which helenalin causes RMS cell death in vitro, with smaller contributions to cell death from mitochondrial and ER stress pathways, and NF-**κ**B p65 deactivation.

## Data Availability

Data are contained within the article and Supplementary Materials.

## Supplementary Materials

The following are available online at https://www.mdpi.com/article/10.3390/ph14121258/s1: Figure S1: The liquid chromatography (LC) analysis of helenalin incubated in PBS at 37 °C under a 5% CO2 atmosphere for 3 days. (A) Representative LC graphs from triplicates at four different time points, where helenalin was detected at a retention time of 5.88–5.94 min. (B) the LC condition. (C) Graphs to show the amount of helenalin at 4 different time points as a% of day 0 control. Data is presented as mean ± standard deviation (SD); Figure S2: Viable cell number assessed using an MTT assay after fibroblast cells were treated with various concentrations (0–10 µM) of helenalin for 24 h and 72 h; Figure S3: Concentration optimisation of NAC for the pre-treatment in RD and RH30 cells. (A) The comparative analysis of ROS levels indicated by RFU of CM-H2DCFDA of RMS cells from the measurement using a plate reader. Here, the cells were treated with helenalin for 24 h after treated with DMSO (Hele *w/o* NAC), 1 mM NAC (Hele + 1 mM NAC), 2 mM NAC (Hele + 2 mM NAC), 3 mM NAC (Hele + 3 mM NAC), 5 mM NAC (Hele + 5 mM NAC) and 10 mM NAC (Hele + 10 mM NAC) for 2 h. (B) The comparative analysis of viabilities of RMS cells from the crystal violet staining assay. Here, the cells were treated with DMSO, 1 mM NAC, 2 mM NAC, 3 mM NAC, 5 mM NAC and 10 mM NAC for 2 h. Significances were tested using one-way ANOVA with Dunnett post hoc tests (** *p* ≤ 0.01, *** *p* ≤ 0.001); Figure S4: Concentration optimisation of ZVAD for the pre-treatment in RD and RH30 cells. (A) The comparative analysis of MMP levels indicated by RFU of TMRM of RMS cells from the measurement using a plate reader. Here, the cells were treated with helenalin for 24 h after treated with DMSO (Hele *w/o* ZVAD), 10 µM ZVAD (Hele + 10 µM ZVAD), 20 µM ZVAD (Hele + 20 µM ZVAD), 40 µM ZVAD (Hele + 40 µM ZVAD), 60 µM ZVAD (Hele + 60 µM ZVAD) and 100 µM ZVAD (Hele + 100 µM ZVAD) for 24 h. (B) The comparative analysis of viabilities of RMS cells from the crystal violet staining assay. Here, the cells were treated with DMSO, 10 µM ZVAD, 20 µM ZVAD, 40 µM ZVAD, 60 µM ZVAD and 100 µM ZVAD for 24 h. Significances were tested using one-way ANOVA with Dunnett post hoc tests (** *p* ≤ 0.01); Figure S5: Concentration optimisation of TUDCA for the pre-treatment in RD and RH30 cells. (A) The comparative analysis of Ca^2+^ levels indicated by RFU of Fluo-4 of RMS cells from the measurement using a plate reader. Here, the cells were treated with helenalin for 24 h after being treated with DMSO (Hele *w/o* TUDCA), 10 µM TUDCA (Hele + 10 µM TUDCA), 20 µM TUDCA (Hele + 20 µM TUDCA), 40 µM TUDCA (Hele + 40 µM TUDCA), 60 µM TUDCA (Hele + 60 µM TUDCA) and 100 µM TUDCA (Hele + 100 µM TUDCA) for 30 min. (B) The comparative analysis of viabilities of RMS cells from the crystal violet staining assay. Here, the cells were treated with DMSO, 10 µM TUDCA, 20 µM TUDCA, 40 µM TUDCA, 60 µM TUDCA and 100 µM TUDCA for 30 min. Significances were tested using one-way ANOVA with Dunnett post hoc tests (* *p* ≤ 0.05, ** *p* ≤ 0.01); Figure S6: Concentration optimisation of TNF-**α** for the pre-treatment in RD and RH30 cells. (A) The comparative analysis of phosphorylated levels of NF-**κ**B p65 at Serine 529 indicated by median fluorescence of PE of RMS cells from the flow cytometry measurement. Here, the cells were treated with helenalin for 24 h after being treated with DMSO (Hele *w/o* TNF), 1 ng/mL TNF-**α** (Hele + 1 ng/mL TNF), 2 ng/mL TNF-**α** (Hele + 2 ng/mL TNF), 5 ng/mL TNF-**α** (Hele + 5 ng/mL TNF) and 10 ng/mL TNF-**α** (Hele + 10 ng/mL TNF) for 1 h. (B) The comparative analysis of viabilities of RMS cells from the crystal violet staining assay. Here, the cells were treated with DMSO, 1 ng/mL TNF-**α** (1 ng/mL TNF), 2 ng/mL TNF-**α** (2 ng/mL TNF), 5 ng/mL TNF-**α** (5 ng/mL TNF) and 10 ng/mL TNF-**α** (10 ng/mL TNF) for 1 h. Significances were tested using one-way ANOVA with Dunnett post hoc tests (* *p* ≤ 0.05, ** *p* ≤ 0.01); Figure S7: Extra cell survival assessment of RMS cells using PI/AV staining. (A) & (C) The flow cytometric measurement of cell viabilities of RD and RH30 cells stained by PI and AV each, and (B) & (D) Comparison of% of PI-/AV- (live) cell populations in RD and RH30 cells each. Cells were treated with DMSO (0 µM helenalin, DMSO) and 5 µM helenalin for 24 h after pre-treatment of DMSO for 24 h (5 µM Hele), both 5 mM NAC for 2 h and 100 µM TUDCA for 24 h (NAC + TUDCA), both 5 mM NAC for 2 h and 100 µM ZVAD for 30 min (NAC + ZVAD), both 5 mM NAC for 2 h and 5 ng/mL TNF-**α** for 1 h (NAC + TNF), both 100 µM TUDCA for 24 h and 100 µM ZVAD for 30 min (TUDCA + ZVAD), both 100 µM TUDCA for 24 h and 5 ng/mL TNF-**α** for 1 h (TUDCA + TNF) and both 100 µM ZVAD for 30 min and 5 ng/mL TNF-**α** for 1 h (ZVAD + TNF). Significances were tested using one-way ANOVA with Tukey post hoc tests (ns *p* > 0.05, * *p* ≤ 0.05, ** (&&) *p* ≤ 0.01).

## References

[1] Barlow J.W., Wiley J.C., Mous M., Narendran A., Gee M.F.W., Goldberg M., Sexsmith E., Malkin D. (2006). Differentiation of Rhabdomyosarcoma Cell Lines Using Retinoic Acid. Pediatric Blood Cancer.

[2] Wang C. (2012). Childhood Rhabdomyosarcoma: Recent Advances and Prospective Views. J. Dent. Res..

[3] Parham D.M., Barr F.G. (2013). Classification of Rhabdomyosarcoma and Its Molecular Basis. Adv. Anat. Pathol..

[4] Arden K.C., Anderson M.J., Finckenstein F.G., Czekay S., Cavenee W.K. (1996). Detection of the t(2;13) Chromosomal Translocation in Alveolar Rhabdomyosarcoma Using the Reverse Transcriptase-Polymerase Chain Reaction. Genes Chromosomes Cancer.

[5] Gryder B.E., Yohe M.E., Chou H.-C., Zhang X., Marques J., Wachtel M., Schaefer B., Sen N., Song Y., Gualtieri A. (2017). PAX3-FOXO1 Establishes Myogenic Super Enhancers and Confers BET Bromodomain Vulnerability. Cancer Discov..

[6] Hinson A.R.P., Jones R., Crose L.E.S., Belyea B.C., Barr F.G., Linardic C.M. (2013). Human Rhabdomyosarcoma Cell Lines for Rhabdomyosarcoma Research: Utility and Pitfalls. Front. Oncol..

[7] Lyss G., Schmidt T.J., Merfort I., Pahl H.L. (1997). Helenalin, an Anti-Inflammatory Sesquiterpene Lactone from Arnica, Selectively Inhibits Transcription Factor NF-KappaB. Biol. Chem..

[8] Zwicker P., Schultze N., Niehs S., Albrecht D., Methling K., Wurster M., Wachlin G., Lalk M., Lindequist U., Haertel B. (2017). Differential Effects of Helenalin, an Anti-Inflammatory Sesquiterpene Lactone, on the Proteome, Metabolome and the Oxidative Stress Response in Several Immune Cell Types. Toxicol. In Vitro.

[9] Lee K.H., Hall I.H., Mar E.C., Starnes C.O., ElGebaly S.A., Waddell T.G., Hadgraft R.I., Ruffner C.G., Weidner I. (1977). Sesquiterpene Antitumor Agents: Inhibitors of Cellular Metabolism. Science.

[10] Huang P.-R., Yeh Y.-M., Wang T.-C.V. (2005). Potent Inhibition of Human Telomerase by Helenalin. Cancer Lett..

[11] Heilmann J., Wasescha M.R., Schmidt T.J. (2001). The Influence of Glutathione and Cysteine Levels on the Cytotoxicity of Helenanolide Type Sesquiterpene Lactones against KB Cells. Bioorganic Med. Chem..

[12] Lyß G., Knorre A., Schmidt T.J., Pahl H.L., Merfort I. (1998). The Anti-Inflammatory Sesquiterpene Lactone Helenalin Inhibits the Transcription Factor NF-ΚB by Directly Targeting P65*. J. Biol. Chem..

[13] Xia Y., Shen S., Verma I.M. (2014). NF-ΚB, an Active Player in Human Cancers. Cancer Immunol. Res..

[14] Singh V., Gupta D., Arora R. (2015). NF-KB as a Key Player in Regulation of Cellular Radiation Responses and Identification of Radiation Countermeasures. Discoveries.

[15] Kim S.H., Danilenko M., Kim T.S. (2008). Differential Enhancement of Leukaemia Cell Differentiation without Elevation of Intracellular Calcium by Plant-Derived Sesquiterpene Lactone Compounds. Br. J. Pharmacol..

[16] Lim C.B., Fu P.Y., Ky N., Zhu H.S., Feng X., Li J., Srinivasan K.G., Hamza M.S., Zhao Y. (2012). NF-ΚB P65 Repression by the Sesquiterpene Lactone, Helenalin, Contributes to the Induction of Autophagy Cell Death. BMC Complementary Altern. Med..

[17] Jang J.H., Iqbal T., Min K.-J., Kim S., Park J.-W., Son E.-I., Lee T.-J., Kwon T.K. (2013). Helenalin-Induced Apoptosis Is Dependent on Production of Reactive Oxygen Species and Independent of Induction of Endoplasmic Reticulum Stress in Renal Cell Carcinoma. Toxicol. In Vitro.

[18] Grippo A.A., Hall I.H., Kiyokawa H., Muraoka O., Shen Y.C., Lee K.H. (1992). The Cytotoxicity of Helenalin, Its Mono and Difunctional Esters, and Related Sesquiterpene Lactones in Murine and Human Tumor Cells. Drug Des. Discov..

[19] Liu J., Zhao Y., Shi Z., Bai Y. (2019). Antitumor Effects of Helenalin in Doxorubicin-Resistant Leukemia Cells Are Mediated via Mitochondrial Mediated Apoptosis, Loss of Mitochondrial Membrane Potential, Inhibition of Cell Migration and Invasion and Downregulation of PI3-Kinase/AKT/m-TOR Signalling Pathway. J. Buon.

[20] Li Y., Zeng Y., Huang Q., Wen S., Wei Y., Chen Y., Zhang X., Bai F., Lu Z., Wei J. (2019). Helenalin from Centipeda Minima Ameliorates Acute Hepatic Injury by Protecting Mitochondria Function, Activating Nrf2 Pathway and Inhibiting NF-ΚB Activation. Biomed. Pharmacother..

[21] Kordi S., Zarghami N., Akbarzadeh A., Rahmati Y.M., Ghasemali S., Barkhordari A., Tozihi M. (2016). A Comparison of the Inhibitory Effect of Nano-Encapsulated Helenalin and Free Helenalin on Telomerase Gene Expression in the Breast Cancer Cell Line, by Real-Time PCR. Artif. Cells Nanomed. Biotechnol..

[22] Potter A.J., Gollahon K.A., Palanca B.J.A., Harbert M.J., Choi Y.M., Moskovitz A.H., Potter J.D., Rabinovitch P.S. (2002). Flow Cytometric Analysis of the Cell Cycle Phase Specificity of DNA Damage Induced by Radiation, Hydrogen Peroxide and Doxorubicin. Carcinogenesis.

[23] Rieger A.M., Nelson K.L., Konowalchuk J.D., Barreda D.R. (2011). Modified Annexin V/Propidium Iodide Apoptosis Assay for Accurate Assessment of Cell Death. J. Vis. Exp..

[24] Richards C.H., Roxburgh C.S.D., Anderson J.H., McKee R.F., Foulis A.K., Horgan P.G., McMillan D.C. (2012). Prognostic Value of Tumour Necrosis and Host Inflammatory Responses in Colorectal Cancer. Br. J. Surg..

[25] Werfel T.A., Elion D.L., Rahman B., Hicks D.J., Sanchez V., Gonzales-Ericsson P.I., Nixon M.J., James J.L., Balko J.M., Scherle P.A. (2019). Treatment-Induced Tumor Cell Apoptosis and Secondary Necrosis Drive Tumor Progression in the Residual Tumor Microenvironment through MerTK and IDO1. Cancer Res..

[26] Papale M., Buccarelli M., Mollinari C., Russo M.A., Pallini R., Ricci-Vitiani L., Tafani M. (2020). Hypoxia, Inflammation and Necrosis as Determinants of Glioblastoma Cancer Stem Cells Progression. Int. J. Mol. Sci..

[27] Beck R., Verrax J., Dejeans N., Taper H., Calderon P.B. (2009). Menadione Reduction by Pharmacological Doses of Ascorbate Induces an Oxidative Stress That Kills Breast Cancer Cells. Int. J. Toxicol..

[28] Halasi M., Wang M., Chavan T.S., Gaponenko V., Hay N., Gartel A.L. (2013). ROS Inhibitor N-Acetyl-l-Cysteine Antagonizes the Activity of Proteasome Inhibitors. Biochem. J..

[29] Chen Y., Azad M.B., Gibson S.B. (2009). Superoxide Is the Major Reactive Oxygen Species Regulating Autophagy. Cell Death Differ..

[30] Lomeli N., Di K., Pearre D.C., Chung T.-F., Bota D.A. (2020). Mitochondrial-Associated Impairments of Temozolomide on Neural Stem/Progenitor Cells and Hippocampal Neurons. Mitochondrion.

[31] Chien C.-H., Hsueh W.-T., Chuang J.-Y., Chang K.-Y. (2021). Dissecting the Mechanism of Temozolomide Resistance and Its Association with the Regulatory Roles of Intracellular Reactive Oxygen Species in Glioblastoma. J. Biomed. Sci..

[32] Benítez-Rangel E., Olguín-Albuerne M., López-Méndez M.C., Domínguez-Macouzet G., Guerrero-Hernández A., Morán J. (2020). Caspase-3 Activation Correlates With the Initial Mitochondrial Membrane Depolarization in Neonatal Cerebellar Granule Neurons. Front. Cell Dev. Biol..

[33] Ricci J.-E., Gottlieb R.A., Green D.R. (2003). Caspase-Mediated Loss of Mitochondrial Function and Generation of Reactive Oxygen Species during Apoptosis. J. Cell Biol..

[34] Li Y., Li J., Huang H., Yang M., Zhuang D., Cheng X., Zhang H., Fu X. (2016). Microcystin-LR Induces Mitochondria-Mediated Apoptosis in Human Bronchial Epithelial Cells. Exp. Ther. Med..

[35] Morrison R., Lodge T., Evidente A., Kiss R., Townley H. (2017). Ophiobolin A, a Sesterpenoid Fungal Phytotoxin, Displays Different Mechanisms of Cell Death in Mammalian Cells Depending upon the Cancer Cell Origin. Int. J. Oncol..

[36] Fabian S.-G., Jose L.G.-G., Mari C.G.-C., Federico V.P. (2014). Mitochondrial Biogenesis in Health and Disease. Molecular and Therapeutic Approaches. Curr. Pharm. Des..

[37] Doherty E., Perl A. (2017). Measurement of Mitochondrial Mass by Flow Cytometry during Oxidative Stress. React. Oxyg. Species.

[38] Wu J., Chen S., Liu H., Zhang Z., Ni Z., Chen J., Yang Z., Nie Y., Fan D. (2018). Tunicamycin Specifically Aggravates ER Stress and Overcomes Chemoresistance in Multidrug-Resistant Gastric Cancer Cells by Inhibiting N-Glycosylation. J. Exp. Clin. Cancer Res..

[39] Yoon Y.M., Lee J.H., Yun S.P., Han Y.-S., Yun C.W., Lee H.J., Noh H., Lee S.-J., Han H.J., Lee S.H. (2016). Tauroursodeoxycholic Acid Reduces ER Stress by Regulating of Akt-Dependent Cellular Prion Protein. Sci. Rep..

[40] Xie Q., Khaoustov V.I., Chung C.C., Sohn J., Krishnan B., Lewis D.E., Yoffe B. (2002). Effect of Tauroursodeoxycholic Acid on Endoplasmic Reticulum Stress-Induced Caspase-12 Activation. Hepatology.

[41] Wang D., Baldwin A.S. (1998). Activation of Nuclear Factor-KappaB-Dependent Transcription by Tumor Necrosis Factor-Alpha Is Mediated through Phosphorylation of RelA/P65 on Serine 529. J. Biol. Chem..

[42] Choi K.-C., Lee Y.-H., Jung M.G., Kwon S.H., Kim M.-J., Jun W.J., Lee J., Lee J.M., Yoon H.-G. (2009). Gallic Acid Suppresses Lipopolysaccharide-Induced Nuclear Factor-ΚB Signaling by Preventing RelA Acetylation in A549 Lung Cancer Cells. Mol. Cancer Res..

[43] Hayden M.S., Ghosh S. (2014). Regulation of NF-ΚB by TNF Family Cytokines. Semin. Immunol..

[44] Perl A., Gergely P., Nagy G., Koncz A., Banki K. (2004). Mitochondrial Hyperpolarization: A Checkpoint of T-Cell Life, Death and Autoimmunity. Trends Immunol..

[45] Miwa S., Yamamoto N., Hayashi K., Takeuchi A., Igarashi K., Tsuchiya H. (2020). Recent Advances and Challenges in the Treatment of Rhabdomyosarcoma. Cancers.

[46] Hoffmann R., von Schwarzenberg K., López-Antón N., Rudy A., Wanner G., Dirsch V.M., Vollmar A.M. (2011). Helenalin Bypasses Bcl-2-Mediated Cell Death Resistance by Inhibiting NF-ΚB and Promoting Reactive Oxygen Species Generation. Biochem. Pharmacol..

[47] Sorg C., Schmid E., Bortel N., Fuchs J., Ellerkamp V. (2021). Antitumor Effects of Curcumin in Pediatric Rhabdomyosarcoma in Combination with Chemotherapy and Phototherapy In Vitro. Int. J. Oncol..

[48] Mu Y., Liu Y., Li L., Tian C., Zhou H., Zhang Q., Yan B. (2015). The Novel Tubulin Polymerization Inhibitor MHPT Exhibits Selective Anti-Tumor Activity against Rhabdomyosarcoma In Vitro and In Vivo. PLoS ONE.

[49] Yuksel S.N., Dikmen M., Canturk Z. (2021). Evaluation of Real Time Cell Proliferation, Anti-Inflammatory and Wound Healing Potential of Helenalin on HaCaT Keratinocytes Treated with Lipopolysaccharide Stimulated Monocytes. Indian J. Pharm. Sci..

[50] DiPaola R.S. (2002). To Arrest or Not to G(2)-M Cell-Cycle Arrest: Commentary Re: A. K. Tyagi et al., Silibinin Strongly Synergizes Human Prostate Carcinoma DU145 Cells to Doxorubicin-Induced Growth Inhibition, G(2)-M Arrest, and Apoptosis. Clin. Cancer Res..

[51] Lu W.-J., Peng W., Sun Q.-Q., Li Y.-H., Chen B., Yu L.-T., Xu Y.-Z., Wang S.-Y., Zhao Y.-L. (2018). #2714, a Novel Active Inhibitor with Potent G2/M Phase Arrest and Antitumor Efficacy in Preclinical Models. Cell Death Discov..

[52] Elmore S. (2007). Apoptosis: A Review of Programmed Cell Death. Toxicol. Pathol..

[53] Chaabane W., User S.D., El-Gazzah M., Jaksik R., Sajjadi E., Rzeszowska-Wolny J., Łos M.J. (2013). Autophagy, Apoptosis, Mitoptosis and Necrosis: Interdependence Between Those Pathways and Effects on Cancer. Arch. Immunol. Ther. Exp..

[54] Guidicelli G., Chaigne-Delalande B., Dilhuydy M.-S., Pinson B., Mahfouf W., Pasquet J.-M., Mahon F.-X., Pourquier P., Moreau J.-F., Legembre P. (2009). The Necrotic Signal Induced by Mycophenolic Acid Overcomes Apoptosis-Resistance in Tumor Cells. PLoS ONE.

[55] Berges C., Fuchs D., Opelz G., Daniel V., Naujokat C. (2009). Helenalin Suppresses Essential Immune Functions of Activated CD4+ T Cells by Multiple Mechanisms. Mol. Immunol..

[56] Gach K., Długosz A., Janecka A. (2015). The Role of Oxidative Stress in Anticancer Activity of Sesquiterpene Lactones. Naunyn-Schmiedeberg’s Arch Pharm..

[57] Valencia A., Morán J. (2004). Reactive Oxygen Species Induce Different Cell Death Mechanisms in Cultured Neurons. Free. Radic. Biol. Med..

[58] Joanna D., Anna J. (2019). Helenalin—A Sesquiterpene Lactone with Multidirectional Activity. Curr. Drug Targets.

[59] Tsujimoto Y., Shimizu S. (2007). Role of the Mitochondrial Membrane Permeability Transition in Cell Death. Apoptosis.

[60] Rasul A., Khan M., Yu B., Ali M., Bo Y.J., Yang H., Ma T. (2013). Isoalantolactone, a Sesquiterpene Lactone, Induces Apoptosis in SGC-7901 Cells via Mitochondrial and Phosphatidylinositol 3-Kinase/Akt Signaling Pathways. Arch. Pharm. Res..

[61] Hamzeloo-Moghadam M., Aghaei M., Fallahian F., Jafari S.M., Dolati M., Abdolmohammadi M.H., Hajiahmadi S., Esmaeili S. (2015). Britannin, a Sesquiterpene Lactone, Inhibits Proliferation and Induces Apoptosis through the Mitochondrial Signaling Pathway in Human Breast Cancer Cells. Tumor Biol..

[62] Chen C.-N., Huang H.-H., Wu C.-L., Lin C.P.C., Hsu J.T.A., Hsieh H.-P., Chuang S.-E., Lai G.-M. (2007). Isocostunolide, a Sesquiterpene Lactone, Induces Mitochondrial Membrane Depolarization and Caspase-Dependent Apoptosis in Human Melanoma Cells. Cancer Lett..

[63] Kohno K., Normington K., Sambrook J., Gething M.J., Mori K. (1993). The Promoter Region of the Yeast KAR2 (BiP) Gene Contains a Regulatory Domain That Responds to the Presence of Unfolded Proteins in the Endoplasmic Reticulum. Mol. Cell. Biol..

[64] Ellgaard L., Ruddock L.W. (2005). The Human Protein Disulphide Isomerase Family: Substrate Interactions and Functional Properties. EMBO Rep..

[65] Høyer-Hansen M., Jäättelä M. (2007). Connecting Endoplasmic Reticulum Stress to Autophagy by Unfolded Protein Response and Calcium. Cell Death Differ..

[66] Zhang Y., Qu P., Ma X., Qiao F., Ma Y., Qing S., Zhang Y., Wang Y., Cui W. (2018). Tauroursodeoxycholic Acid (TUDCA) Alleviates Endoplasmic Reticulum Stress of Nuclear Donor Cells under Serum Starvation. PLoS ONE.

[67] Fang B., Wen S., Li Y., Bai F., Wei Y., Xiong Y., Huang Q., Lin X. (2021). Prediction and Verification of Target of Helenalin against Hepatic Stellate Cell Activation Based on MiR-200a-Mediated PI3K/Akt and NF-ΚB Pathways. Int. Immunopharmacol..

[68] Mitomo K., Nakayama K., Fujimoto K., Sun X., Seki S., Yamamoto K. (1994). Two Different Cellular Redox Systems Regulate the DNA-Binding Activity of the P50 Subunit of NF-Kappa B In Vitro. Gene.

[69] Rüngeler P., Castro V., Mora G., Gören N., Vichnewski W., Pahl H.L., Merfort I., Schmidt T.J. (1999). Inhibition of Transcription Factor NF-ΚB by Sesquiterpene Lactones: A Proposed Molecular Mechanism of Action. Bioorganic Med. Chem..

[70] Morgan M.J., Liu Z. (2011). Crosstalk of Reactive Oxygen Species and NF-ΚB Signaling. Cell Res..

[71] Mosmann T. (1983). Rapid Colorimetric Assay for Cellular Growth and Survival: Application to Proliferation and Cytotoxicity Assays. J. Immunol. Methods.

[72] Sulen A., Gullaksen S.-E., Bader L., McClymont D.W., Skavland J., Gavasso S., Gjertsen B.T. (2016). Signaling Effects of Sodium Hydrosulfide in Healthy Donor Peripheral Blood Mononuclear Cells. Pharmacol. Res..

