# Supplementary material for: Mechanism of Action of the Sesquiterpene Compound Helenalin in Rhabdomyosarcoma Cells
Source: Pharmaceuticals (Basel). 2021 Dec 2;14(12):1258. doi: 10.3390/ph14121258 (PMC8703838; doi:10.3390/ph14121258)
Supplement: Supplementary file 1 [file pharmaceuticals-14-01258-s001.zip › pharmaceuticals-1404326-supplementary.pdf]

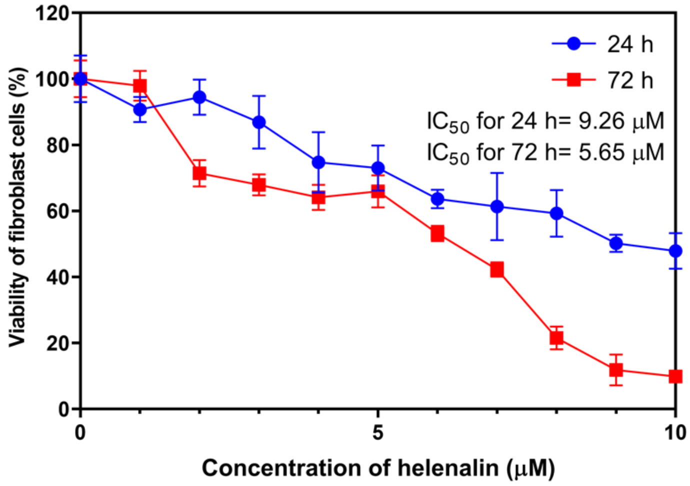

Figure S1. Viable cell number assessed using an MTT assay after fibroblast cells were treated with various concentrations (0 - 10 µM) of helenalin for 24 h and 72 h.

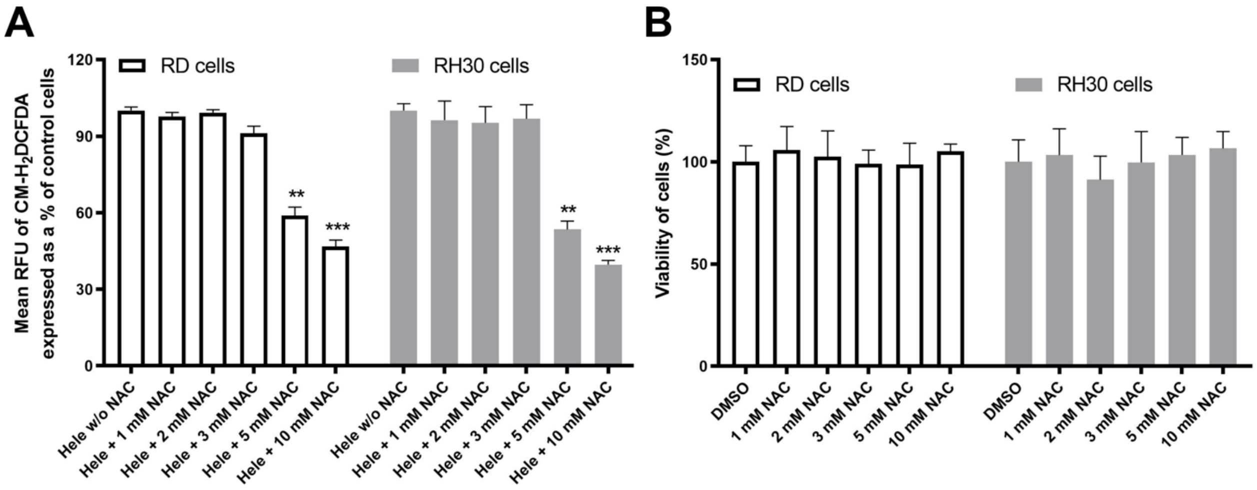

Figure S2. Concentration optimisation of NAC for the pre-treatment in RD and RH30 cells. (A) The comparative analysis of ROS levels indicated by RFU of CM-H2DCFDA of RMS cells from the measurement using a plate reader. Here, the cells were treated with helenalin for 24 h after treated with DMSO (Hele w/o NAC), 1 mM NAC (Hele + 1 mM NAC), 2 mM NAC (Hele + 2 mM NAC), 3 mM NAC (Hele + 3 mM NAC), 5 mM NAC (Hele + 5 mM NAC) and 10 mM NAC (Hele + 10 mM NAC) for 2 h. (B) The comparative analysis of viabilities of RMS cells from the crystal violet staining assay. Here, the cells were treated with DMSO, 1 mM NAC, 2 mM NAC, 3 mM NAC, 5 mM NAC and 10 mM NAC for 2 h. Significances were tested using a two-tailed t-test (** P≤0.01, *** P≤0.001).

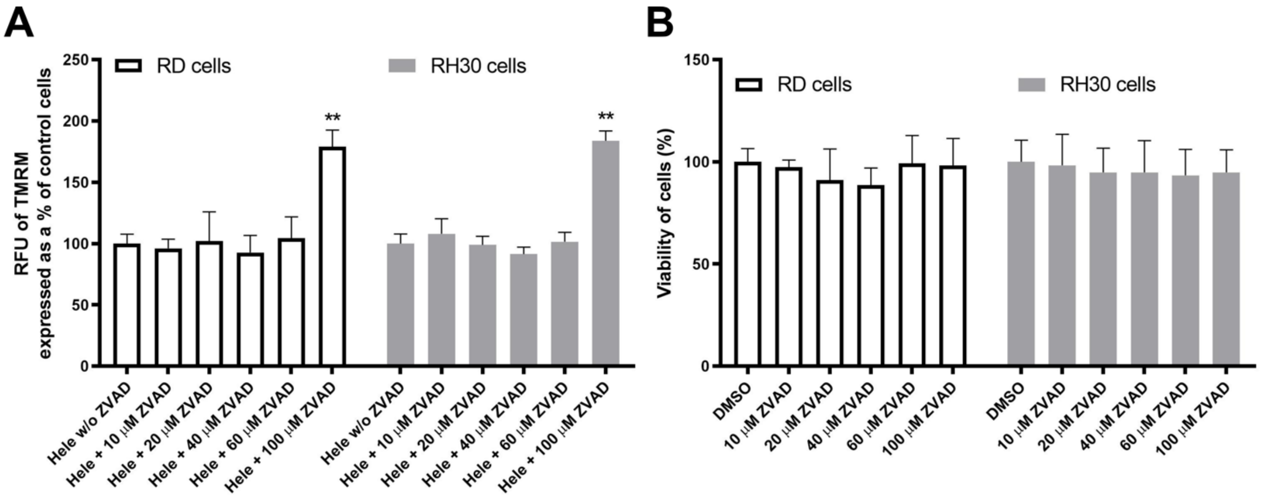

Figure S3. Concentration optimisation of ZVAD for the pre-treatment in RD and RH30 cells. (A) The comparative analysis of MMP levels indicated by RFU of TMRM of RMS cells from the measurement using a plate reader. Here, the cells were treated with helenalin for 24 h after treated with DMSO (Hele w/o ZVAD), 10 µM ZVAD (Hele + 10 µM ZVAD), 20 µM ZVAD (Hele + 20 µM ZVAD), 40 µM ZVAD (Hele + 40 µM ZVAD), 60 µM ZVAD (Hele + 60 µM ZVAD) and 100 µM ZVAD (Hele + 100 µM ZVAD) for 24 h. (B) The comparative analysis of viabilities of RMS cells from the crystal violet staining assay. Here, the cells were treated with DMSO, 10 µM ZVAD, 20 µM ZVAD, 40 µM ZVAD, 60 µM ZVAD and 100 µM ZVAD for 24 h. Significances were tested using a two-tailed t-test (** P≤0.01).

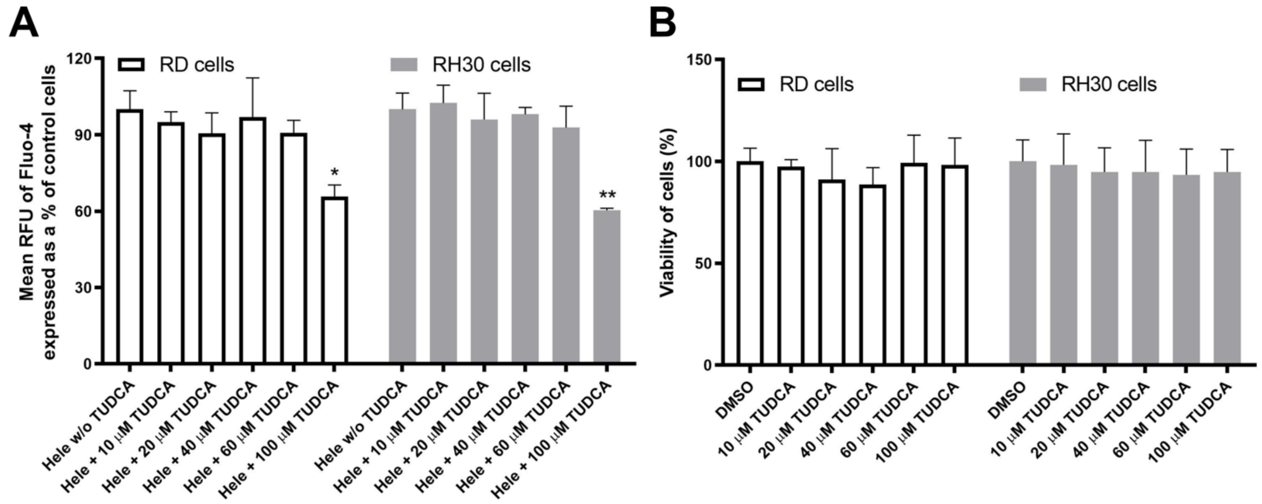

Figure S4. Concentration optimisation of TUDCA for the pre-treatment in RD and RH30 cells. (A) The comparative analysis of Ca^{2+} levels indicated by RFU of Fluo-4 of RMS cells from the measurement using a plate reader. Here, the cells were treated with helenalin for 24 h after treated with DMSO (Hele w/o TUDCA), 10 µM TUDCA (Hele + 10 µM TUDCA), 20 µM TUDCA (Hele + 20 µM TUDCA), 40 µM TUDCA (Hele + 40 µM TUDCA), 60 µM TUDCA (Hele + 60 µM TUDCA) and 100 µM TUDCA (Hele + 100 µM TUDCA) for 30 min. (B) The comparative analysis of viabilities of RMS cells from the crystal violet staining assay. Here, the cells were treated with DMSO, 10 µM TUDCA, 20 µM TUDCA, 40 µM TUDCA, 60 µM TUDCA and 100 µM TUDCA for 30 min. Significances were tested using a two-tailed t-test (* P≤0.05, ** P≤0.01).

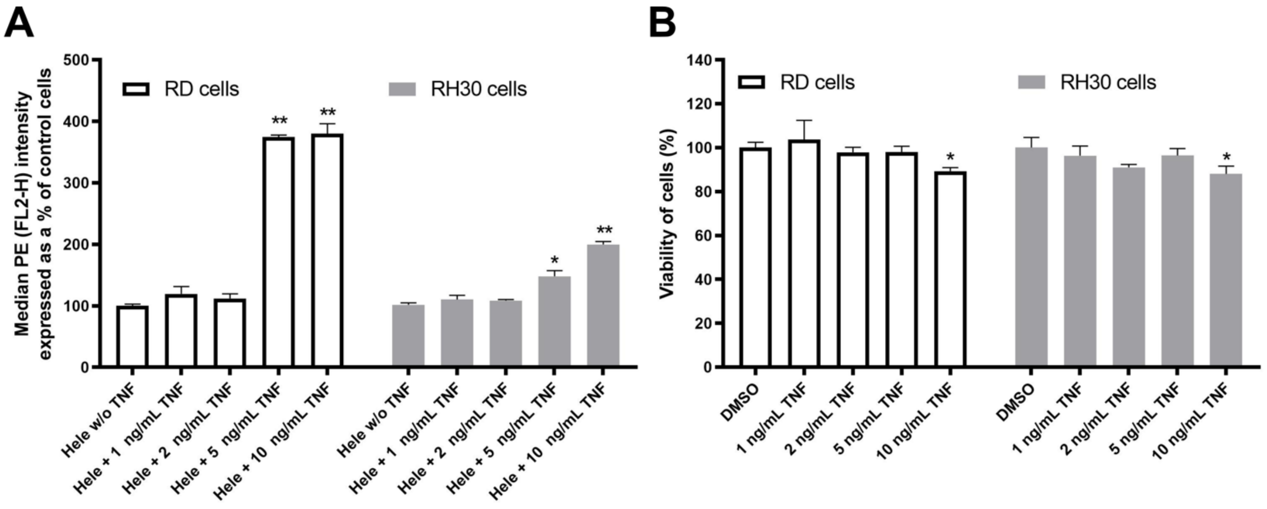

Figure S5. Concentration optimisation of TNF-α for the pre-treatment in RD and RH30 cells. (A) The comparative analysis of phosphorylated levels of NF-κB p65 at Serine 529 indicated by median fluorescence of PE of RMS cells from the flow cytometry measurement. Here, the cells were treated with helenalin for 24 h after treated with DMSO (Hele w/o TNF), 1 ng/mL TNF-α (Hele + 1 ng/mL TNF), 2 ng/mL TNF-α (Hele + 2 ng/mL TNF), 5 ng/mL TNF-α (Hele + 5 ng/mL TNF) and 10 ng/mL TNF-α (Hele + 10 ng/mL TNF) for 1 h. (B) The comparative analysis of viabilities of RMS cells from the crystal violet staining assay. Here, the cells were treated with DMSO, 1 ng/mL TNF-α (1 ng/mL TNF), 2 ng/mL TNF-α (2 ng/mL TNF), 5 ng/mL TNF-α (5 ng/mL TNF) and 10 ng/mL TNF-α (10 ng/mL TNF) for 1 h. Significances were tested using a two-tailed t-test (* $P \leq 0.05$, ** $P \leq 0.01$).

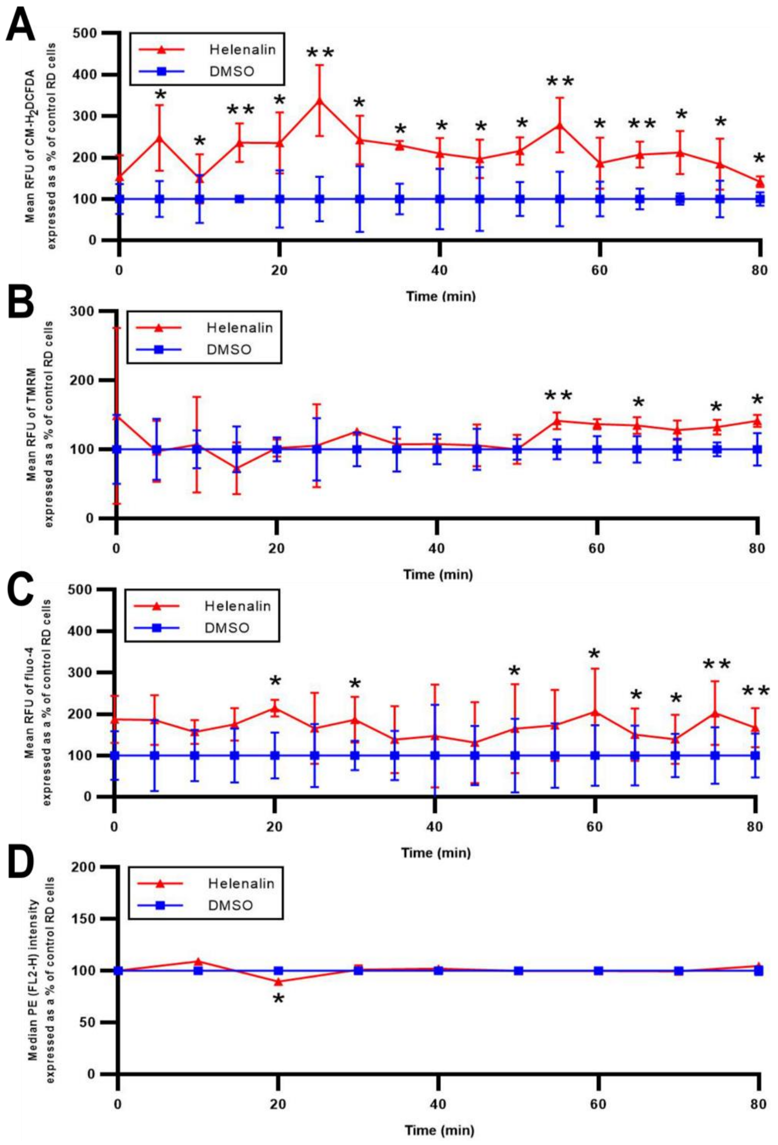

Figure S6. Signal changes from several pathways by helenalin treatment in RD cells. (A) ROS levels (mean RFU of CM-H_2DCFDA) of cells treated with DMSO and 5 µM helenalin over 80 min, (B) MMP levels (mean RFU of TMRM) of cells treated with DMSO and 5 µM helenalin over 80 min, (C) Ca^{2+} levels (mean RFU of Fluo-4) of cells treated with DMSO and 5 µM helenalin over 80 min and (D) NF-**κ**B p65 phosphorylation levels (mean RFU of PE) of cells treated with DMSO and 5 µM helenalin over 80 min. Significances were tested using a two-tailed t-test (* P≤0.05, ** P≤0.01).

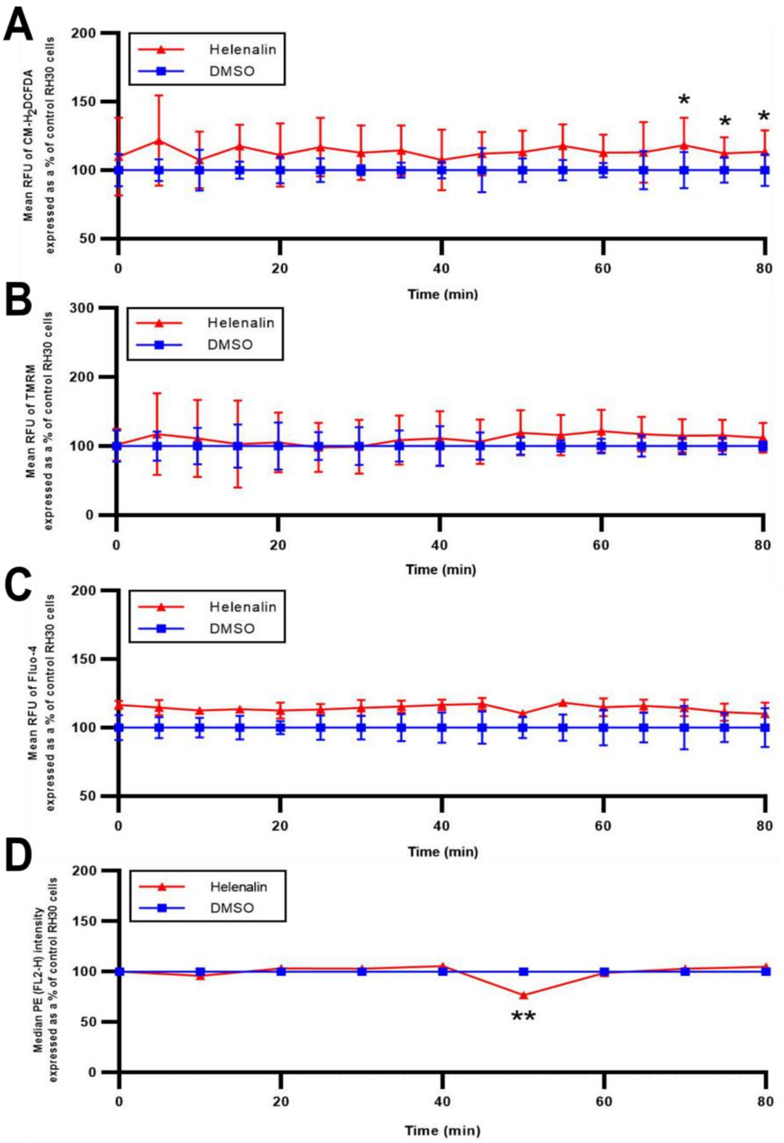

Figure S7. Signal changes from several pathways by helenalin treatment in RH30 cells. (A) ROS levels (mean RFU of CM-H_2DCFDA) of cells treated with DMSO and 5 µM helenalin over 80 min, (B) MMP levels (mean RFU of TMRM) of cells treated with DMSO and 5 µM helenalin over 80 min, (C) Ca^{2+} levels (mean RFU of Fluo-4) of cells treated with DMSO and 5 µM helenalin over 80 min and (D) NF-**κ**B p65 phosphorylation levels (mean RFU of PE) of cells treated with DMSO and 5 µM helenalin over 80 min. Significances were tested using a two-tailed t-test (* P≤0.05, ** P≤0.01).
